# Capture of mechanically interlocked molecules by rhodium-mediated terminal alkyne dimerisation†

**DOI:** 10.1039/d4ra00566j

**Published:** 2024-03-05

**Authors:** Thomas M. Hood, Samantha Lau, Adrian B. Chaplin

**Affiliations:** a Department of Chemistry, University of Warwick Gibbet Hill Road Coventry CV4 7AL UK tom.m.hood@warwick.ac.uk a.b.chaplin@warwick.ac.uk

## Abstract

The transition metal-mediated dimerisation of terminal alkynes is an attractive and atom-economic method for preparing conjugated 1,3-enynes. Using a phosphine-based macrocyclic pincer ligand, we demonstrate how this transformation can be extended to the synthesis of novel, hydrocarbon-based interlocked molecules: a rotaxane by ‘active’ metal template synthesis and a catenane by sequential ‘active’ and ‘passive’ metal template procedures.

## Abstract 2

Active metal template synthesis of an enyne-based rotaxane and catenane.

## Graphical abstract

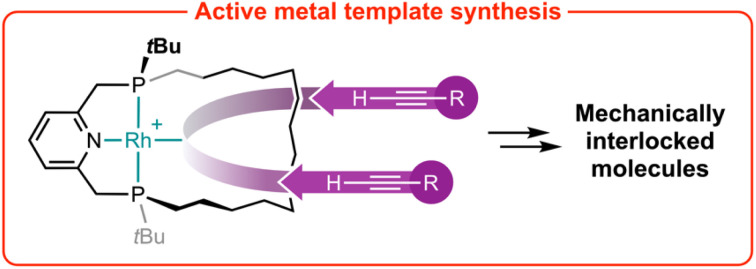

## Introduction

**Fig. 1:**
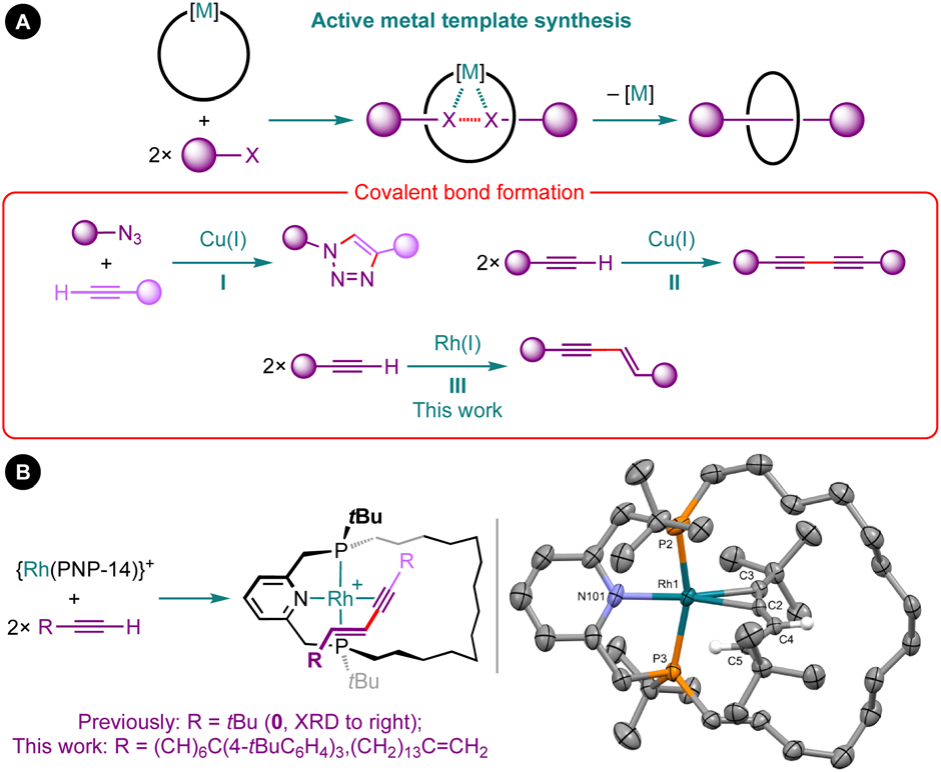
(A) Active metal template synthesis of interlocked molecules and (B) terminal alkyne dimerisation reactions promoted by macrocyclic rhodium(i) PNP pincer complexes. Solid-state structure of complex 0 shown with thermal ellipsoids drawn at 30% probability and most H-atoms omitted.

Coordination chemistry is a prominent feature of contemporary methods for constructing mechanically interlocked molecules, such as rotaxanes and catenanes.^1^ Active metal template synthesis has emerged as a particularly effective strategy, exploiting a metal to position and covalently fuse the precursor fragments together in an entangled arrangement (Fig. 1A).^2^ Whilst this strategy has been successfully implemented using a variety of metal-mediated transformations, the overwhelming majority of examples are based on copper-mediated alkyne–azide cycloaddition reactions (I) or Glaser couplings (II) in combination with bidentate nitrogen-based macrocycles.^2,3^

**Fig. 2:**
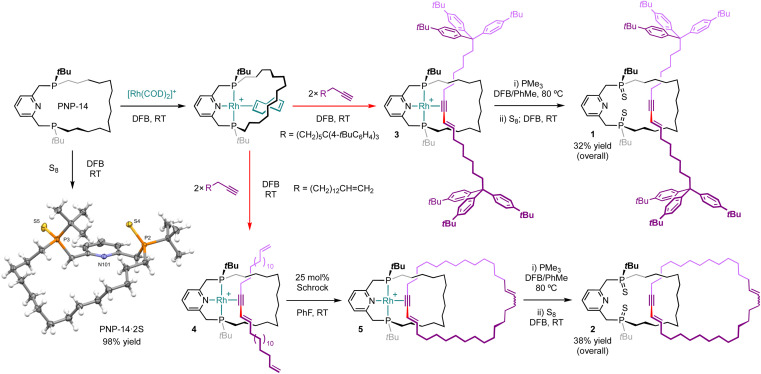
Synthesis of rotaxane 1 and catenane 2. [B(3,5-(CF_3_)_2_C_6_H_3_)_4_]^−^ counterions omitted for clarity and solid-state structure of PNP-14·2S shown with thermal ellipsoids drawn at 50% probability.

As part of our work exploring the organometallic chemistry of terminal alkyne dimerisation reactions^4^ promoted by macrocyclic pincer complexes (Fig. 1B),^5,6^ we speculated that this transformation could be adapted into an active metal template procedure for the preparation of mechanically interlocked 1,3-enynes (III). We herein describe the preparation of hydrocarbon-based rotaxane 1 and catenane 2 derived from the phosphine-based macrocyclic pincer ligand PNP-14 (Fig. 2).^7^ Despite the widespread use of phosphine ligands in organotransition metal chemistry and homogenous catalysis,^8^ this constitutes the first application in active metal template synthesis of mechanically interlocked molecules.

## Results and discussion

Using a protocol developed previously for rhodium(i) *E*-enyne complex 0 (Fig. 1B),^6,9^ rotaxanate 3 and pseudo-rotaxanate 4 were obtained in quantitative spectroscopic yield upon treatment of [Rh(PNP-14)(*η*^2^-COD)]^+^ (COD = cyclooctadiene; *δ*_31P_ 57.4/45.9, ^2^*J*_PP_ = 312 Hz, ^1^*J*_RhP_ ∼135 Hz) with the novel bulky aryl stoppered terminal alkyne HC≡C(CH_2_)_6_C(4-*t*BuC_6_H_4_)_3_ and methylene-spaced ene–yne HC≡C(CH_2_)_13_CH=CH_2_, respectively, in the weakly coordinating solvent 1,2-difluorobenzene (DFB) at room temperature (Fig. 2).^10^ The new rhodium derivatives present ^1^H NMR resonances at *δ* 6.95/5.94 (3) and 7.01/5.98 (4) with ^3^*J*_HH_ coupling constants of ∼15 Hz that are diagnostic for coordinated *E*-enynes, whilst the *C*_1_ symmetry of the molecules is manifested in the observation of inequivalent ^31^P NMR resonances at *δ* 58.6/54.9 (3) and 56.9/53.1 (4) that are coupled to ^103^Rh (^1^*J*_RhP_ = 128 Hz) and display *trans*^2^*J*_PP_ coupling constants of ∼393 Hz.^11^ Subsequent treatment of 4 with 25 mol% Schrock's catalyst (CAS: 139220-25-0) at room temperature enabled capture of catenate 5 by ring closing olefin metathesis, with complete conversion confirmed after monitoring the reaction *in situ* for 5 days at room temperature using ^1^H and ^31^P{^1^H} NMR spectroscopy and (tandem) ESI-MS.

Removal of rhodium from 3 and 5 was achieved by heating with excess PMe_3_ (20 equiv.) to give the corresponding rotaxane (1′, *δ*_31P_ 2.42/0.92) and catenane (2′, *δ*_31P_ 1.46/0.78), alongside [Rh(PMe_3_)_4_]^+^ as the rhodium-containing by-product.^12^ To facilitate isolation, 1′ and 2′ were converted into the corresponding phosphine sulphides 1 and 2 by treatment with S_8_ which were thereafter obtained in 32% and 38% overall yield (from PNP-14) following purification on silica. Formation of the new interlocked molecules was corroborated by analysis of isolated materials using NMR spectroscopy and ESI-MS. Threading of the enyne breaks the *C*_2_ symmetry of the macrocycle and this desymmetrisation is evident in both the ^1^H and ^31^P{^1^H} NMR spectra of 1 (*δ*_31P_ 63.71/63.65) and 2 (*δ*_31P_ 63.6/63.5), alongside perturbation of the macrocycle resonances relative to independently isolated PNP-14·2S (*δ*_31P_ 64.7, NMR stack plots provided in the ESI†). The interlocked nature of 1 and 2 was also substantiated by high resolution tandem mass spectrometry experiments,^13^ whereby selective fragmentation of the [M + H]^+^ ions (1, 1584.1321, calcd 1584.1339 *m*/*z*; 2, 982.7566, calcd 982.7549 *m*/*z*) gave the [M + H]^+^ ion of PNP-14·2S (542.3154/542.3159, calcd 542.3167 *m*/*z*) as the major species in both cases.

## Conclusions

These results serve as proof of principle for the application of transition mediated terminal alkyne dimerisation reactions in the synthesis of mechanically interlocked molecules, whilst also demonstrating how phosphine-based functional groups can be integrated into the structure of these intriguing molecules.

## Experimental section

All manipulations were performed under an atmosphere of argon using Schlenk and glove box techniques unless otherwise stated. Glassware was oven dried at 150 °C overnight and flame-dried under vacuum prior to use. Molecular sieves were activated by heating at 300 °C *in vacuo* overnight. Fluorobenzene and 1,2-difluorobenzene (DFB) were pre-dried over Al_2_O_3_, distilled from calcium hydride and dried twice over 3 Å molecular sieves.^10^ CD_2_Cl_2_ was freeze–pump–thaw degassed and dried over 3 Å molecular sieves. THF and dioxane were distilled from sodium/benzophenone and stored over 3 Å molecular sieves. DMSO was freeze–pump–thaw degassed and dried up to five times and finally stored over 3 Å molecular sieves. SiMe_4_ was distilled from liquid Na/K alloy and stored over a potassium mirror. Other anhydrous solvents were purchased from Acros Organics or Sigma-Aldrich, freeze–pump–thaw degassed and stored over 3 Å molecular sieves. PMe_3_ in toluene and 1,6-dibromohexane were freeze–pump–thawed degassed and dried twice over 3 Å molecular sieves before use. Schrock's catalyst (CAS: 139220-25-0) was recrystallised from SiMe_4_ at −30 °C before use. *n*BuLi was titrated before use.^14^ Br(CH_2_)_6_C(4-*t*BuC_6_H_4_)_3_,^15^ 15-bromo-1-pentadecene,^16^ [Rh(COD)_2_][BAr^F^_4_],^17^ and PNP-14 ^7^ were synthesized according to published procedures. All other reagents are commercial products and were used as received. NMR spectra were recorded on Bruker spectrometers under argon at 298 K unless otherwise stated. Chemical shifts are quoted in ppm and coupling constants in Hz. NMR spectra in DFB were recorded using an internal capillary of C_6_D_6_. High resolution (HR) ESI-MS and MS/MS were recorded on Bruker Maxis Plus instrument. Microanalysis was performed at the London Metropolitan University by Stephen Boyer.

### Preparation of HC≡C(CH_2_)_6_C(4-*t*BuC_6_H_4_)_3_

A suspension of Br(CH_2_)_6_C(4-*t*BuC_6_H_4_)_3_ (290 mg, 504 μmol) in DMSO (5 mL) was treated dropwise with THF until homogeneous. A suspension of HC≡CLi·H_2_N(CH_2_)_2_NH_2_ (51.0 mg, 554 μmol) in THF (5 mL) was then added and the resulting suspension heated at 130 °C for 48 h. The reaction was quenched by addition of H_2_O (2 mL) and the product extracted into CH_2_Cl_2_ (3 × 5 mL). The combined organic extracts were washed with brine (2 × 10 mL), dried over MgSO_4_ and then concentrated *in vacuo* to give an oily white solid. Purification using a silica plug (hexane → 1 : 1 hexane : CH_2_Cl_2_) afforded the product as a white solid. Yield: 220 mg (422 μmol, 84%; mp. 142–143 °C).

^1^H NMR (500 MHz, CDCl_3_): *δ* 7.24 (d, ^3^*J*_HH_ = 8.4, 6H, *m*-Ar), 7.14 (d, ^3^*J*_HH_ = 8.4, 6H, *o*-Ar), 2.48–2.52 (m, 2H, Ar_3_CCH̲_2_), 2.13 (td, ^3^*J*_HH_ = 7.1, ^4^*J*_HH_ = 2.6, 2H, CH̲_2_C≡CH), 1.91 (t, ^4^*J*_HH_ = 2.6, 1H, C≡CH), 1.44 (pent, ^3^*J*_HH_ = 7.1, 2H, CH̲_2_CH_2_C≡CH), 1.25–1.36 (m, 4H, 2×CH_2_), 1.30 (s, 27H, *t*Bu), 1.05–1.12 (m, 2H, CH_2_).

^13^C{^1^H} NMR (126 MHz, CDCl_3_): *δ* 148.2 (s, *t*BuC̲), 145.0 (s, *i*-Ar), 129.0 (s, *o*-Ar), 124.5 (s, *m*-Ar), 84.9 (s, C̲≡CH), 68.2 (s, C≡C̲H), 55.5, (s, Ar_3_C̲), 40.7 (s, Ar_3_CC̲H_2_), 34.4 (s, *t*Bu{C}), 31.5 (s, *t*Bu{CH_3_}), 30.1 (s, CH_2_), 28.8 (s, CH_2_), 28.7 (s, C̲H_2_CH_2_C≡CH), 25.7 (s, CH_2_), 18.5 (s, C̲H_2_C≡CH).

HR ESI-MS (positive ion 4 kV): 559.3684 ([M + K]^+^, calcd 559.3701) *m*/*z*.

### Preparation of HC≡C(CH_2_)_13_CH=CH_2_

A suspension of 15-bromo-1-pentadecene (1.22 g, 4.24 mmol) and HC≡CLi·H_2_N(CH_2_)_2_NH_2_ (0.41 g, 4.45 mmol) in 1,4-dioxane-DMSO (10 : 5 mL) was stirred at 120 °C for 16 h. The reaction was quenched by addition of H_2_O (15 mL) and product extracted into hexane (3 × 15 mL). The combined organic extracts were washed with brine (2 × 10 mL), dried over MgSO_4_ and then concentrated *in vacuo* to give an off-white oily wax. The analytically pure compound was obtained as a colourless wax by repeated column chromatography (silica, hexane; *R*_f_ = 0.37). Yield: 244 mg (1.04 mmol, 25%; mp. 43–48 °C).

^1^H NMR (500 MHz, CDCl_3_): *δ* 5.81 (ddt, ^3^*J*_HH_ = 16.9, 10.2, 6.7, 1H, H_2_C=CH̲), 4.99 (ddt, ^3^*J*_HH_ = 16.9, ^2^*J*_HH_ = 2.2, ^4^*J*_HH_ = 1.6, 1H, H̲_2_C=CH), 4.93 (ddt, ^3^*J*_HH_ = 10.2, ^2^*J*_HH_ = 2.2, ^4^*J*_HH_ = 1.3, 1H, H̲_2_C=CH), 2.18 (td, ^3^*J*_HH_ = 7.1, ^4^*J*_HH_ = 2.6, 2H, CH̲_2_C≡CH), 2.01–2.07 (m, 2H, H_2_C=CHCH̲_2_), 1.94 (t, ^4^*J*_HH_ = 2.6, 1H, C≡CH̲), 1.52 (pent, ^3^*J*_HH_ = 7.6, 2H, CH̲_2_CH_2_C≡CH), 1.33–1.43 (m, 4H, 2×CH_2_), 1.22–1.33 (m, 16H, 8×CH_2_).

^13^C{^1^H} NMR (126 MHz, CDCl_3_): *δ* 139.4 (s, H_2_C=C̲H), 114.2 (s, H_2_C̲=CH), 85.0 (s, C̲CH), 68.2 (s, C≡C̲H), 34.0 (s, CH_2_=CHC̲H_2_), 29.80 (s, 2×CH_2_), 29.76 (s, 2×CH_2_), 29.7 (s, 2×CH_2_), 29.31 (s, CH_2_), 29.27 (s, CH_2_), 29.1 (s, CH_2_), 28.9 (s, CH_2_), 28.7 (s, CH_2_), 18.6 (s, C̲H_2_C≡CH).

Anal. calcd for C_17_H_30_ (234.43 g mol^−1^): C, 87.10; H, 12.90; N, 0.00. Found: C, 86.99; H, 13.02; N, 0.00.

### Preparation of rotaxane 1

A solution of [Rh(PNP-14)(*η*^2^-COD)][BAr^F^_4_] (8.3 μmol, generated *in situ* from [Rh(COD)_2_][BAr^F^_4_] and PNP-14 as previously described^9^) in DFB (0.5 mL) was treated with HC≡C(CH_2_)_6_C(4-*t*BuC_6_H_4_)_3_ (9.5 mg, 18.2 μmol) and stirred at RT for 5 min. Volatiles were removed *in vacuo* to afford 3 as an orange foam upon removal of volatiles. Crude 3 was then dissolved in DFB (330 μL) and treated with a solution of PMe_3_ in toluene (170 μL, 1 M, 170 μmol) and the resulting solution heated at 80 °C for 5 days. Volatiles were removed *in vacuo* and the residue extracted with hexane. The resulting orange oil was treated with S_8_ (12.6 mg, 49.1 μmol) in DFB (0.5 mL) and stirred at RT for 16 h. Finally, removal of the volatiles *in vacuo* and purification by preparative TLC (silica; 9 : 1 CH_2_Cl_2_ : MeOH; *R*_f_ = 0.4–0.5) afforded the product as a white solid. Yield: 4.2 mg (2.7 μmol, 32%; mp 112 °C).

### Data for 3

^1^H NMR (500 MHz, CD_2_Cl_2_, selected data): *δ* 7.76 (t, ^3^*J*_HH_ = 7.9, 1H, *p*-py), 7.33 (d, ^3^*J*_HH_ = 7.9, 1H, *m*-py), 7.31 (d, ^3^*J*_HH_ = 7.9, 1H, *m*-py), 6.95 (dt, ^3^*J*_HH_ = 14.6, ^3^*J*_HH_ = 6.9, 1H, C≡CCH=CH̲), 5.94 (d, ^3^*J*_HH_ = 15.3, 1H, C≡CC̲H=CH), 1.31 (s, 12H, *t*BuC), 1.30 (s, 38H, *t*BuC), 0.93 (d, ^3^*J*_PH_ = 12.3, 9H, P*t*Bu), 0.89 (d, ^3^*J*_PH_ = 12.3, 9H, P*t*Bu).

^31^P{^1^H} NMR (162 MHz, CD_2_Cl_2_): *δ* 58.6 (dd, ^2^*J*_PP_ = 394, ^1^*J*_RhP_ = 129, 1P), 54.9 (dd, ^2^*J*_PP_ = 394, ^1^*J*_RhP_ = 127, 1P).

^1^H NMR (400 MHz, DFB, selected data): *δ* 7.55 (t, ^3^*J*_HH_ = 8.0, 1H, *p*-py), 5.99 (d, ^3^*J*_HH_ = 15.0, 1H, C≡CCH̲=CH), 1.12–1.17 (m, 54H, *t*BuC), 0.82–0.89 (m, 18H, P*t*Bu).

^31^P{^1^H} NMR (162 MHz, DFB): *δ* 56.1 (dd, ^2^*J*_PP_ = 393, ^1^*J*_RhP_ = 129, 1P), 52.5 (dd, ^2^*J*_PP_ = 393, ^1^*J*_RhP_ = 127, 1P).

### Data for 1′

^31^P{^1^H} NMR (162 MHz, PhF, selected data): *δ* 2.42 (s, 1P), 0.92 (s, 1P).

### Data for 1

^1^H NMR (500 MHz, CDCl_3_): *δ* 7.45 (d, ^3^*J*_HH_ = 7.6, 1H, *m*-py), 7.41 (t, ^3^*J*_HH_ = 7.6, 1H, *p*-py), 7.24 (d, ^3^*J*_HH_ = 8.6, 6H, *m*-Ar), 7.23 (d, ^3^*J*_HH_ = 8.6, 6H, *m*-Ar), 7.13 (d, ^3^*J*_HH_ = 8.3, 12H, 2×*o*-Ar), 7.12 (obscured, 1H, *m*-py), 6.17 (dt, ^2^*J*_HH_ = 16.0, ^3^*J*_HH_ = 6.9, 1H, C≡CCH=CH̲), 5.95 (dt, ^2^*J*_HH_ = 16.0, ^4^*J*_HH_ = 2.0, 1H, C≡CCH̲=CH), 3.84 (app t, ^2^*J*_PH_ = ^2^*J*_HH_ = 13.9, 1H, pyCH̲_2_), 3.60 (dd, ^2^*J*_HH_ = 14.1, ^2^*J*_PH_ = 11.1, 1H, pyCH̲_2_), 3.37 (app t, ^2^*J*_PH_ = ^2^*J*_HH_ = 13.7, 1H, pyCH̲_2_), 3.35 (dd, ^2^*J*_HH_ = 13.8, ^2^*J*_PH_ = 11.3, 1H, pyCH̲_2_), 2.45–2.53 (m, 4H, 2×Ar_3_CCH̲_2_), 2.18–2.28 (m, 1H, CH̲_2_C≡CCH=CH), 2.02–2.16 (m, 4H, C≡CCH=CHCH̲_2_ [*δ* 2.11, 2H] + PCH_2_ [*δ* 2.08, 1H] + CH̲_2_C≡CCH=CH [*δ* 2.07, 1H]), 1.80–1.94 (m, 5H, CH_2_), 1.11–1.68 (m, 34H, CH_2_), 1.292 (s, 27H, *t*BuC), 1.290 (s, 27H, *t*BuC), 1.24 (d, ^3^*J*_PH_ = 15.6, 18H, 2×P*t*Bu), 0.98–1.11 (m, 4H, 2×Ar_3_CCH_2_CH̲_2_).

^13^C{^1^H} NMR (126 MHz, CDCl_3_): *δ* 153.6 (dd, ^2^*J*_PC_ = 6, ^4^*J*_PC_ = 1, *o*-py), 153.5 (d, ^2^*J*_PC_ = 7, *o*-py), 148.2, (s, *t*BuC̲), 148.1 (s, *t*BuC̲), 145.03 (s, *i*-Ar), 144.97 (s, *i*-Ar), 143.4 (s, C≡CCH=C̲H), 135.6 (s, *p*-py), 128.96 (s, *o*-Ar), 128.95 (s, *o*-Ar), 124.53 (s, *m*-Ar), 124.50 (s, *m*-Ar), 123.4 (br, *m*-py), 123.3 (br, *m*-py), 113.3 (s, C≡CC̲H=CH), 92.3 (s, C̲≡CCH=CH), 82.1 (s, C≡C̲CH=CH), 55.43 (s, Ar_3_C̲), 55.39 (s, Ar_3_C̲), 40.83 (s, Ar_3_CC̲H_2_), 40.80 (s, Ar_3_CC̲H_2_), 37.1 (d, ^1^*J*_PC_ = 42, pyC̲H_2_), 36.3 (d, ^1^*J*_PC_ = 41, pyC̲H_2_), 35.3 (d, ^1^*J*_PC_ = 47, P*t*Bu{C}), 35.2 (d, ^1^*J*_PC_ = 47, P*t*Bu{C}), 34.4 (s, 2×*t*BuC̲{C}), 33.3 (s, C≡CCH=CHC̲H_2_), 31.6 (s, 2×*t*BuC̲{CH_3_}), 31.2 (d, ^2^*J*_PC_ = 15, 2×CH_2_), 30.8 (s, CH_2_), 30.6 (s, CH_2_), 28.8–30.0 (m, 12×CH_2_), 28.2 (d, ^1^*J*_PC_ = 48, PCH_2_), 27.7 (d, ^1^*J*_PC_ = 47, PCH_2_), 26.1 (s, 2×Ar_3_CCH_2_C̲H_2_), 25.8 (s, 2×P*t*Bu{CH_3_}), 23.8 (d, ^3^*J*_PC_ = 4, CH_2_), 23.2 (d, ^3^*J*_PC_ = 4, CH_2_), 21.0 (s, C̲H_2_C≡CCH=CH).

^31^P{^1^H} NMR (162 MHz, CDCl_3_): *δ* 63.71 (s, 1P), 63.65 (s, 1P).

HR ESI-MS (positive ion, 4 kV): 1584.1321 ([M + H]^+^, calcd 1584.1339) *m*/*z*.

HR ESI-MS/MS (positive ion; 120 eV @ +1584): 542.3154 ([PNP-14·2S + H]^+^, calcd 542.3167) *m*/*z*.

### Preparation of catenane 2

A solution of [Rh(PNP-14)(*η*^2^-COD)][BAr^F^_4_] (10.7 μmol, generated *in situ* from [Rh(COD)_2_][BAr^F^_4_] and PNP-14 as previously described^9^) in DFB (0.5 mL) was treated with HC≡C(CH_2_)_13_CH=CH_2_ (194 μL, 116 mM, 22.5 μmol) and stirred at RT for 5 min. Volatiles were removed *in vacuo* to afford 4 as an orange oil. Crude 4 was dissolved in fluorobenzene (10 mL) and treated with 25 mol% Schrock's catalyst in 5 mol% portions (0.4 mg, 0.52 μmol) daily over 5 days and periodically assayed by ESI-MS. Volatiles were removed *in vacuo* to afford 5 as an orange oil. Crude 5 was then dissolved in DFB (300 μL) and treated with a solution of PMe_3_ in toluene (200 μL, 1 M, 200 μmol) and the resulting solution heated at 80 °C for 5 days. Volatiles were removed *in vacuo* and the residue extracted with hexane. The resulting orange oil was treated with S_8_ (12.6 mg, 49.1 μmol) in DFB (0.5 mL) and stirred at RT for 16 h. Finally, removal of the volatiles *in vacuo* and purification by preparative TLC (silica; 9 : 1 CH_2_Cl_2_ : MeOH; *R*_f_ = 0.4–0.5) afforded the product as a colourless oil. Yield: 3.7 mg (3.8 μmol, 38%).

### Data for 4

^1^H NMR (500 MHz, CD_2_Cl_2_, selected data): *δ* 7.77 (t, ^3^*J*_HH_ = 7.8, 1H, *p*-py), 7.36 (overlapping d, ^3^*J*_HH_ = 7.9, 2H, *m*-py), 7.01 (dt, ^3^*J*_HH_ = 14.6, ^3^*J*_HH_ = 6.9, 1H, C≡CCH=CH̲), 5.98 (d, ^3^*J*_HH_ = 15.3, 1H, C≡CCH̲=CH), 5.82 (ddt, ^3^*J*_HH_ = 16.8, ^3^*J*_HH_ = 9.8, ^3^*J*_HH_ = 6.7, 2H, CH̲=CH_2_), 4.98 (d, *J*_HH_ = 17, 2H, CH=CH̲_2_), 4.91 (d, *J*_HH_ = 10, 2H, CH=CH̲_2_), 3.37–3.51 (m, 4H, pyCH̲_2_), 0.97 (d, ^3^*J*_PH_ = 12.3, 9H, *t*Bu), 0.91 (d, ^3^*J*_PH_ = 12.2, 9H, *t*Bu).

^31^P{^1^H} NMR (121 MHz, CD_2_Cl_2_): *δ* 56.9 (dd, ^2^*J*_PP_ = 394, ^1^*J*_RhP_ = 129, 1P), 53.1 (dd, ^2^*J*_PP_ = 394, ^1^*J*_RhP_ = 127, 1P).

^1^H NMR (400 MHz, DFB, selected data): *δ* 8.09–8.15 (m, 8H, Ar^F^), 7.53 (obscured t, ^3^*J*_HH_ = 8.2, p-py), 7.49 (br, 4H, Ar^F^), 6.04 (d, ^3^*J*_HH_ = 15.4, 1H, C≡CCH̲=CH), 5.82 (dt, ^3^*J*_HH_ = 15.4, ^3^*J*_HH_ = 8.3, CH̲=CH_2_), 4.80–4.98 (m, 2H, CH=CH̲_2_), 0.87 (app t, *J*_PH_ = 12.8, 18H, *t*Bu).

^31^P{^1^H} NMR (121 MHz, DFB): *δ* 56.5 (dd, ^2^*J*_PP_ = 394, ^1^*J*_RhP_ = 128, 1P), 52.1 (dd, ^2^*J*_PP_ = 394, ^1^*J*_RhP_ = 127, 1P).

HR ESI-MS (positive ion, 4 kV): 1048.7587, ([M]^+^, calcd 1048.7398) *m*/*z*.

### Data for 5

^1^H NMR (400 MHz, CD_2_Cl_2_, selected data): *δ* 7.79 (t, ^3^*J*_HH_ = 8.3, 1H, *p*-py), 7.36 (br d, ^3^*J*_HH_ = 7.3, 2H, *m*-py), 7.03 (br, 1H, C≡CCH=CH̲), 6.00 (d, ^3^*J*_HH_ = 16, 1H, C≡CCH̲=CH), 5.39 (br, 2H, CH=CH), 3.43 (br, 4H, pyCH̲_2_), 0.98 (d, ^3^*J*_PH_ = 11, 9H, P*t*Bu), 0.92 (d, ^3^*J*_PH_ = 12, 9H, P*t*Bu).

^31^P{^1^H} NMR (121 MHz, CD_2_Cl_2_): *δ* 56.7 (d, ^2^*J*_PP_ = 394, ^1^*J*_RhP_ = 129, 1P), 53.3 (d, ^2^*J*_PP_ = 393, ^1^*J*_RhP_ = 127, 1P).

^1^H NMR (400 MHz, DFB, selected data): *δ* 8.09–8.15 (m, 8H, Ar^F^), 7.49 (br, 4H, Ar^F^), 6.01 (br d, ^3^*J*_HH_ = 14.4, 1H, C≡CCH̲=CH), 5.35 (br, 2H, CH=CH), 3.30 (br, 4H, pyCH̲_2_), 0.88 (br d, ^3^*J*_PH_ = 12, 9H, *t*Bu), 0.83 (br d, ^3^*J*_PH_ = 10, 9H, *t*Bu).

^31^P{^1^H} NMR (121 MHz, DFB): *δ* 56.6 (d, ^2^*J*_PP_ = 394, ^1^*J*_RhP_ = 129, 1P), 52.5 (d, ^2^*J*_PP_ = 393, ^1^*J*_RhP_ = 127, 1P).

HR ESI-MS (positive ion, 4 kV): 1020.7092, ([M]^+^, calcd 1020.7085) *m*/*z*.

HR ESI-MS2 (positive ion, 70 eV @ +1020): 578.2543 ([{Rh(PNP-14)}-H_2_]^+^, calcd 578.2546) *m*/*z*.

### Data for 2′

^31^P{^1^H} NMR (162 MHz, DFB, selected data): *δ* 1.46 (s, 1P), 0.78 (s, 1P).

### Data for 2

^1^H NMR (500 MHz, CDCl_3_): *δ* 7.45–7.54 (m, 2H, py), 7.20 (br d, ^3^*J*_HH_ = 5.2, 1H, py), 6.22 (dt, ^3^*J*_HH_ = 16.0, ^3^*J*_HH_ = 7.0, 1H, C≡CCH=CH̲), 5.98 (dt, ^3^*J*_HH_ = 16.1, ^4^*J*_HH_ = 2.0, 1H, C≡CCH̲=CH), 5.36–5.39 (m, 2H, CH=CH), 3.88 (app t, *J*_PH_ = *J*_HH_ = 14, 1H, pyCH̲_2_), 3.59–3.69 (m, 1H, CH_2_), 3.41 (app t, *J*_PH_ = *J*_HH_ = 13, 2H, pyCH̲_2_), 2.04–2.41 (m, 6H, CH_2_), 1.89–1.99 (m, 9H, CH_2_), 1.38–1.47 (obscured m, ∼16H, CH_2_), 1.25–1.35 (m, ∼65H, CH_2_ + P*t*Bu).

^13^C{^1^H} NMR (126 MHz, CDCl_3_): *δ* 153.5–153.8 (m, py), 143.3 (s, C≡CCH=C̲H), 135.7 (s, py), 130.54 (s, CH=CH), 130.46 (s, CH=CH), 123.4 (s, py), 123.3 (s, py), 113.6 (s, C≡CC̲H=CH), 92.5 (s, C̲≡CCH=CH), 82.1 (s, C≡C̲CH=CH), 37.2 (d, ^1^*J*_PC_ = 42, pyC̲H_2_), 36.5 (d, ^1^*J*_PC_ = 41, pyC̲H_2_), 35.5 (d, ^1^*J*_PC_ = 23, P*t*Bu{C}), 35.1 (d, ^1^*J*_PC_ = 24, P*t*Bu{C}), 32.8 (s, CH_2_), 32.1 (s, CH_2_), 32.4 (s, CH_2_), 31.2 (s, CH_2_), 29.9 (s, CH_2_), 29.2–29.8 (m, multiple CH_2_), 29.2 (s, CH_2_), 29.12 (s, CH_2_), 29.09 (s, CH_2_), 29.07 (s, CH_2_), 28.88 (s, CH_2_), 28.86 (s, CH_2_), 28.7 (s, CH_2_), 28.3 (s, CH_2_), 27.9 (s, CH_2_), 27.6 (s, CH_2_), 25.77 (s, P*t*Bu{CH_3_}), 25.75 (s, P*t*Bu{CH_3_}), 23.8 (d, ^3^*J*_PC_ = 4, CH_2_), 23.3 (d, ^3^*J*_PC_ = 4, CH_2_), 22.9 (s, CH_2_), 20.8 (s, CH_2_).

^31^P{^1^H} NMR (121 MHz, CDCl_3_): *δ* 63.6 (s, 1P), 63.5 (s, 1P).

HR ESI-MS (positive ion, 4 kV): 982.7566, ([M + H]^+^, calcd 982.7549) *m*/*z*.

HR ESI-MS2 (positive ion, 60 eV @ +982): 542.3159 ([PNP-14·2S + H]^+^, calcd 542.3167) *m*/*z*.

### Preparation of PNP-14·2S

A solution of PNP-14 (8.5 mg, 17.8 μmol) in DFB (0.5 mL) was treated with S_8_ (1.2 mg, 4.68 μmol) and stirred at RT for 16 h. Volatiles were removed, and the resulting residue washed with methanol (2 × 0.5 mL) and then dried *in vacuo* to afford the product as a white microcrystalline solid. Yield: 9.4 mg (17.3 μmol, 97%; mp. 139–140 °C).

^1^H NMR (500 MHz, CDCl_3_): *δ* 7.59 (t, ^3^*J*_HH_ = 7.8, 1H, *p*-py), 7.35 (d app t, ^3^*J*_HH_ = 7.8, *J*_PH_ = 2, 2H, *m*-py), 3.49 (app t, ^2^*J*_PH_ = ^2^*J*_HH_ = 13, 2H, pyCH̲_2_), 3.43 (app t, ^2^*J*_PH_ = ^2^*J*_HH_ = 14, 2H, pyCH̲_2_), 1.99–2.10 (m, 2H, PCH_2_), 1.68–1.86 (m, 4H, PCH_2_ [*δ* 1.80, 2H] + CH_2_), 1.24–1.58 (m, 22H, CH_2_), 1.17 (d, ^3^*J*_PH_ = 15.9, 18H, *t*Bu).

^13^C{^1^H} NMR (126 MHz, CDCl_3_): *δ* 153.7 (dd, ^2^*J*_PC_ = 8, ^4^*J*_PC_ = 2, *o*-py), 136.6 (t, ^4^*J*_PC_ = 2, *p*-py), 123.4 (app t, *J*_PC_ = 3, *m*-py), 38.9 (d, ^1^*J*_PC_ = 39, pyC̲H_2_), 34.8 (d, ^1^*J*_PC_ = 47, *t*Bu{C}), 30.2 (d, ^2^*J*_PC_ = 15, CH_2_), 27.9 (s, CH_2_), 27.80 (s, CH_2_), 27.79 (s, CH_2_), 27.7 (s, CH_2_), 26.3 (d, ^1^*J*_PC_ = 47, PCH_2_), 25.3 (s, *t*Bu{CH_3_}), 22.4 (d, ^3^*J*_PC_ = 4, CH_2_).

^31^P{^1^H} NMR (162 MHz, CDCl_3_): *δ* 64.7 (s).

HR ESI-MS (positive ion, 4 kV): 542.3160 ([M + H]^+^, calcd 542.3167) *m*/*z*.

## Conflicts of interest

There are no conflicts to declare.

## Supplementary Material

RA-014-D4RA00566J-s001

RA-014-D4RA00566J-s002
