# Supplementary material for: Capture of mechanically interlocked molecules by rhodium-mediated terminal alkyne dimerisation
Source: RSC Adv. 2024 Mar 5;14(11):7740–4. doi: 10.1039/d4ra00566j (PMC10914095; doi:10.1039/d4ra00566j)
Supplement: RA-014-D4RA00566J-s001 [file RA-014-D4RA00566J-s001.pdf]

Electronic Supplementary Material (ESI) for RSC Advances.
This journal is © The Royal Society of Chemistry 2024

Electronic supporting information for

Capture of mechanically interlocked molecules by rhodium-mediated terminal alkyne homocoupling

Thomas M. Hood,* Samantha Lau, and Adrian Chaplin*

Table of contents

1. Preparation of HC≡C$(CH_2)_6$C(4-*t*BuC_6H_4)$_3$ 2
2. Preparation of HC≡C$(CH_2)_{13}$CH=CH_2 3
3. Preparation of rotaxane **1** 4
4. Preparation of catenane **2** 8
5. Preparation of PNP-14·2S 15
6. Comparison of NMR spectra 18

1. Preparation of HC≡C$(CH_2)_6$C(4-*t*BuC_6H_4)$_3$

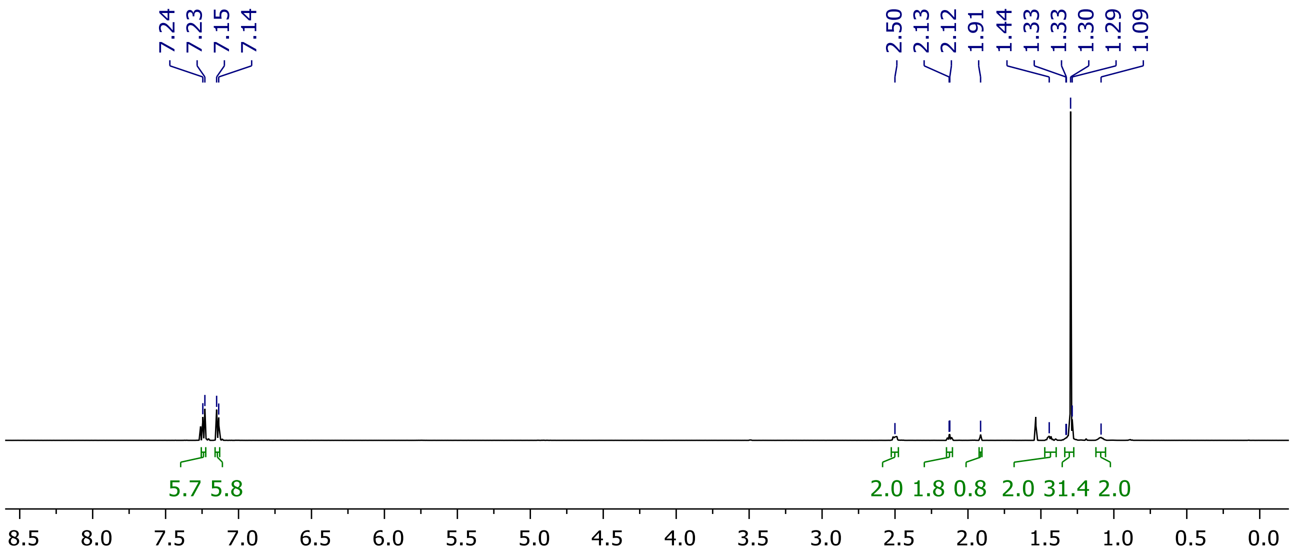

Figure S1. ^{1}H NMR spectrum of HC≡C$(CH_2)_6$C(4-*t*BuC_6H_4)$_3$ ($CDCl_3$, 500 MHz).

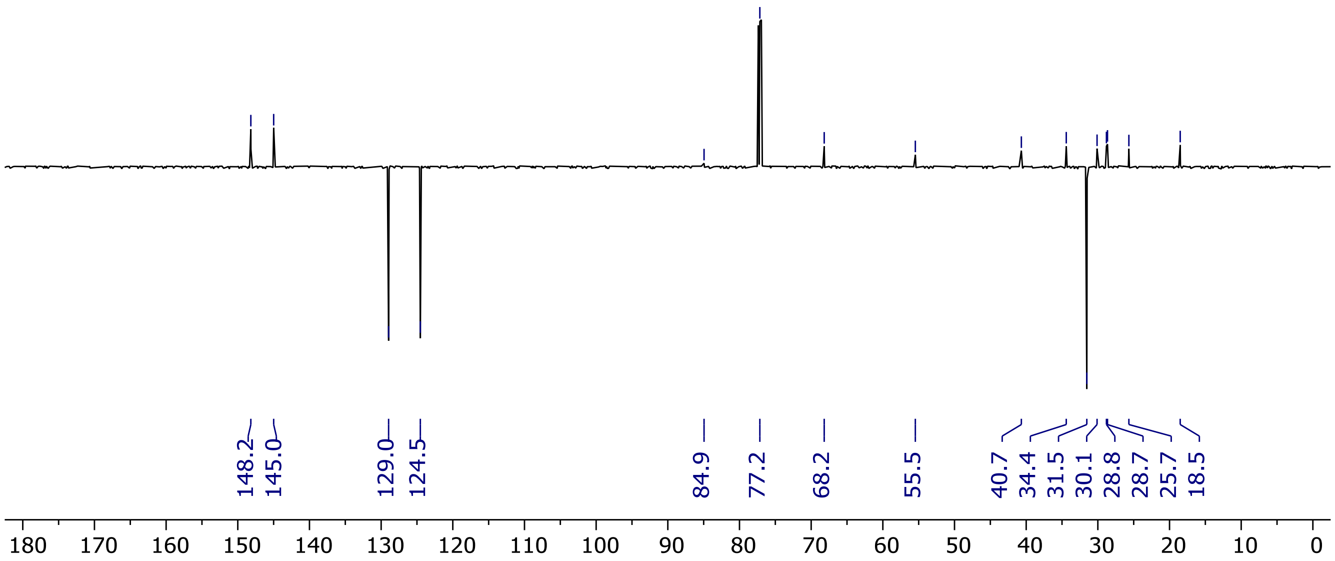

Figure S2. $^{13}C\{^1H\}$ APT NMR spectrum of HC≡C$(CH_2)_6$C(4-*t*BuC_6H_4)$_3$ ($CDCl_3$, 126 MHz).

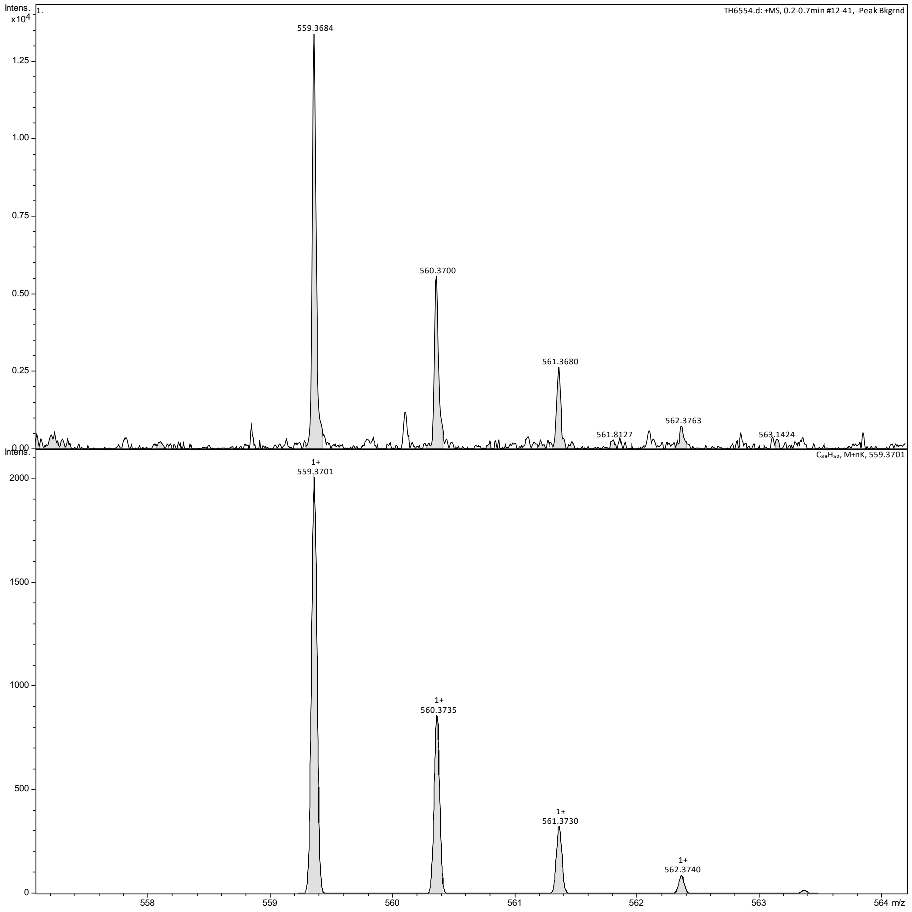

Figure S3. HR ESI-MS of $HC{\equiv}C(CH_2)_6C(4\text{-}t\text{BuC}_6H_4)_3$ (top: observed, bottom: calcd).

2. Preparation of $HC{\equiv}C(CH_2)_{13}CH{=}CH_2$

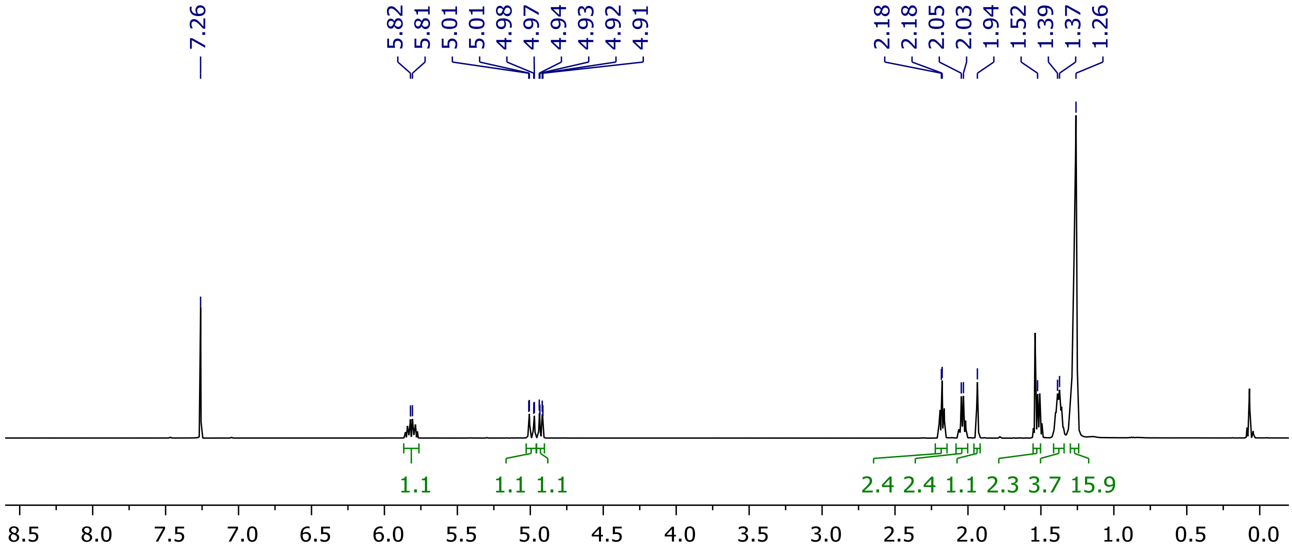

Figure S4. ^{1}H NMR spectrum of $HC{\equiv}C(CH_2)_{13}CH{=}CH_2$ ($CDCl_3$, 500 MHz).

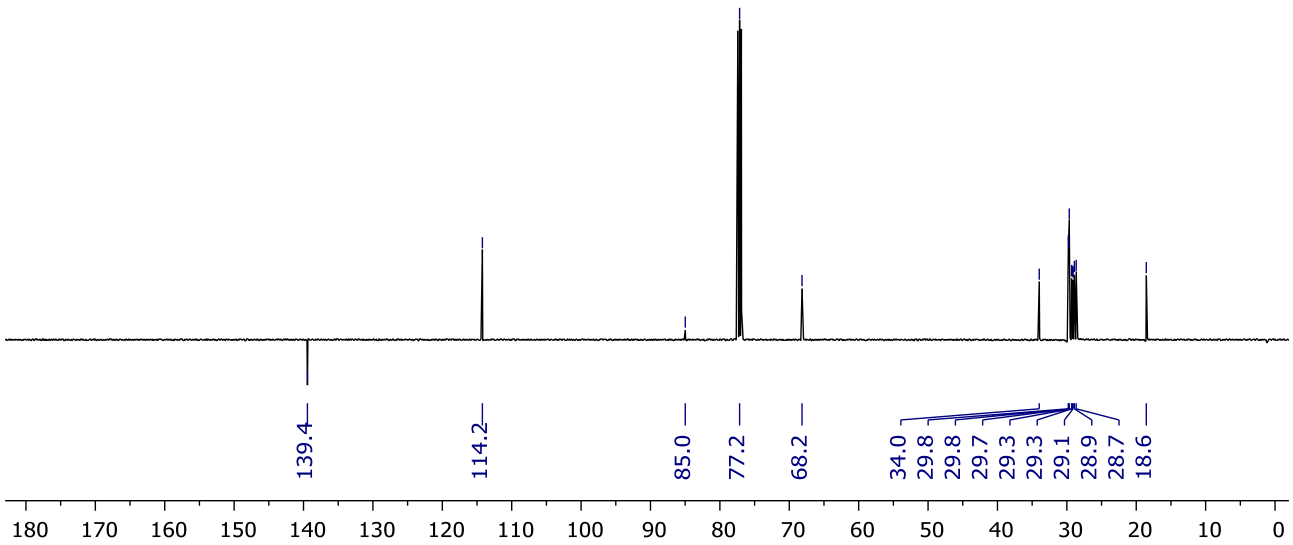

Figure S5. $^{13}C\{^1H\}$ APT NMR spectrum of $HC\equiv C(CH_2)_{13}CH=CH_2$ ($CDCl_3$, 126 MHz).

3. Preparation of rotaxane 1

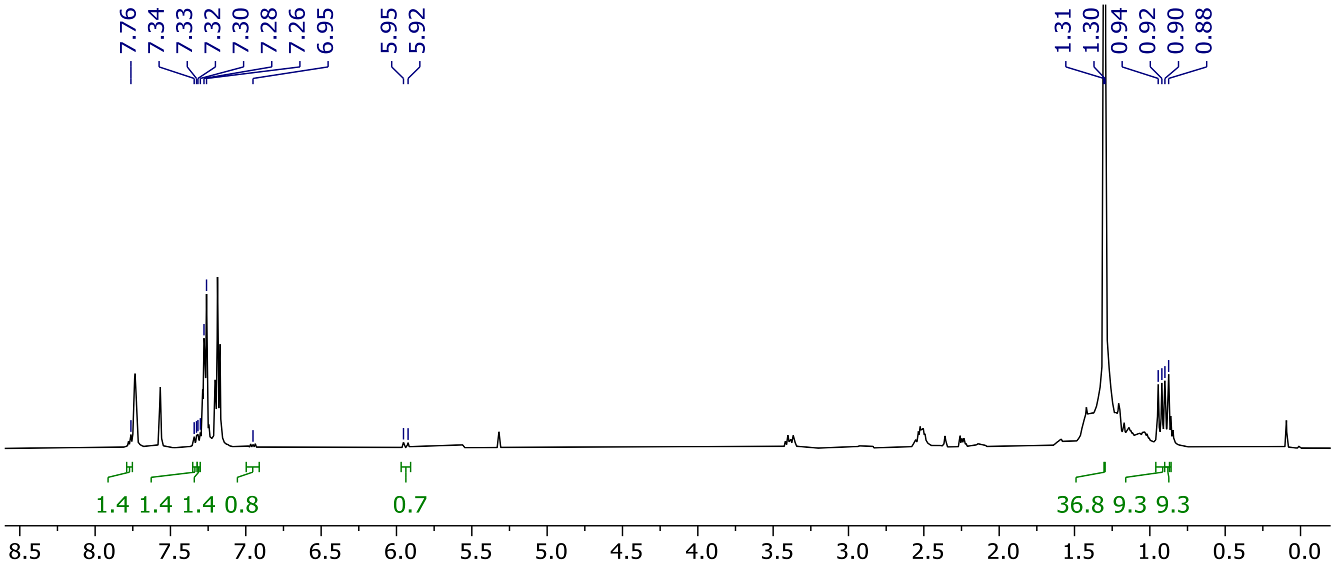

Figure S6. ^1H NMR spectrum of **3** (CD_2Cl_2, 500 MHz).

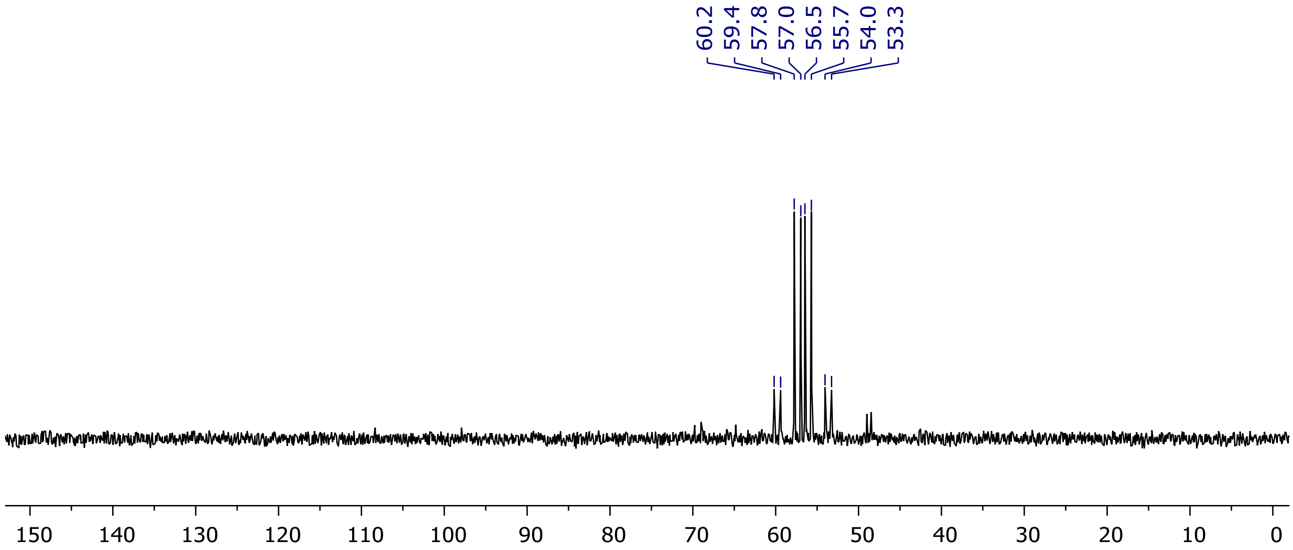

Figure S7. $^{31}P\{^1H\}$ NMR spectrum of **3** (CD_2Cl_2, 162 MHz).

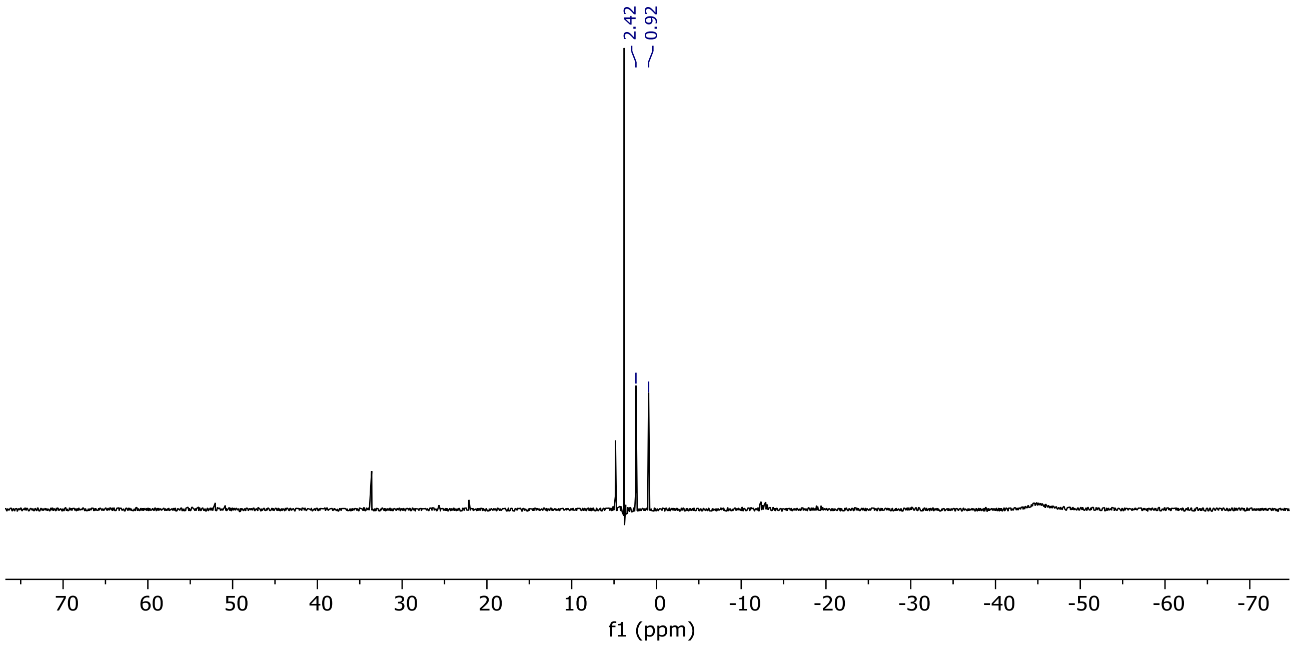

Figure S8. $^{31}P\{^1H\}$ NMR spectrum of **1′** (PhF, 162 MHz).

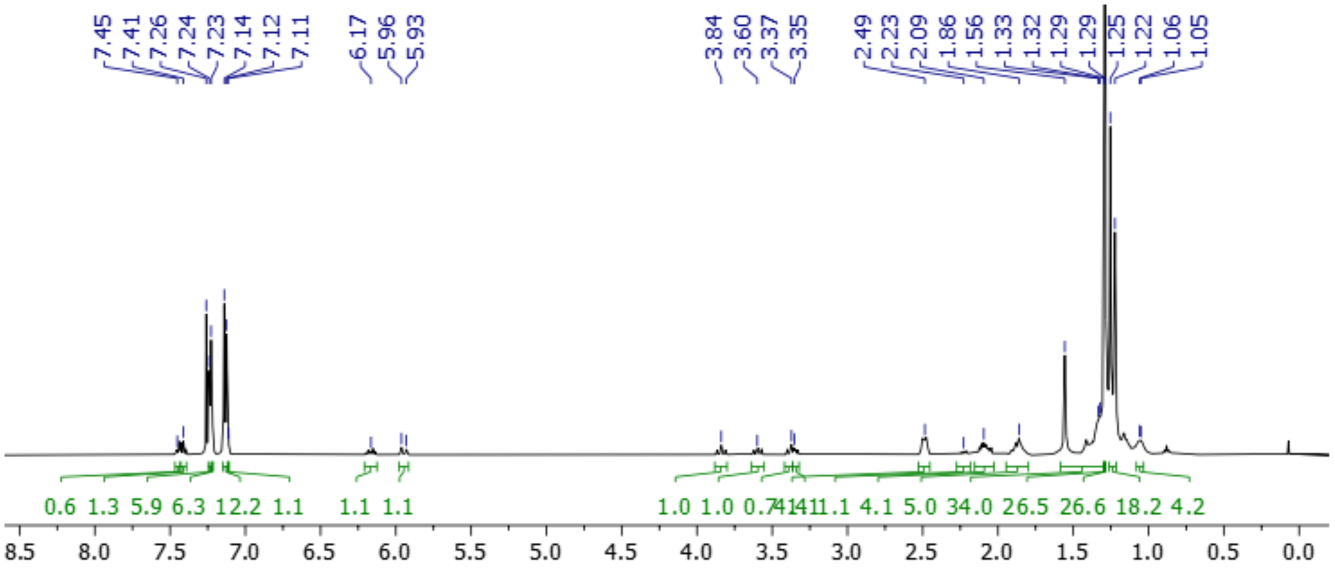

Figure S9. ^{1}H NMR spectrum of **1** ($CDCl_3$, 500 MHz).

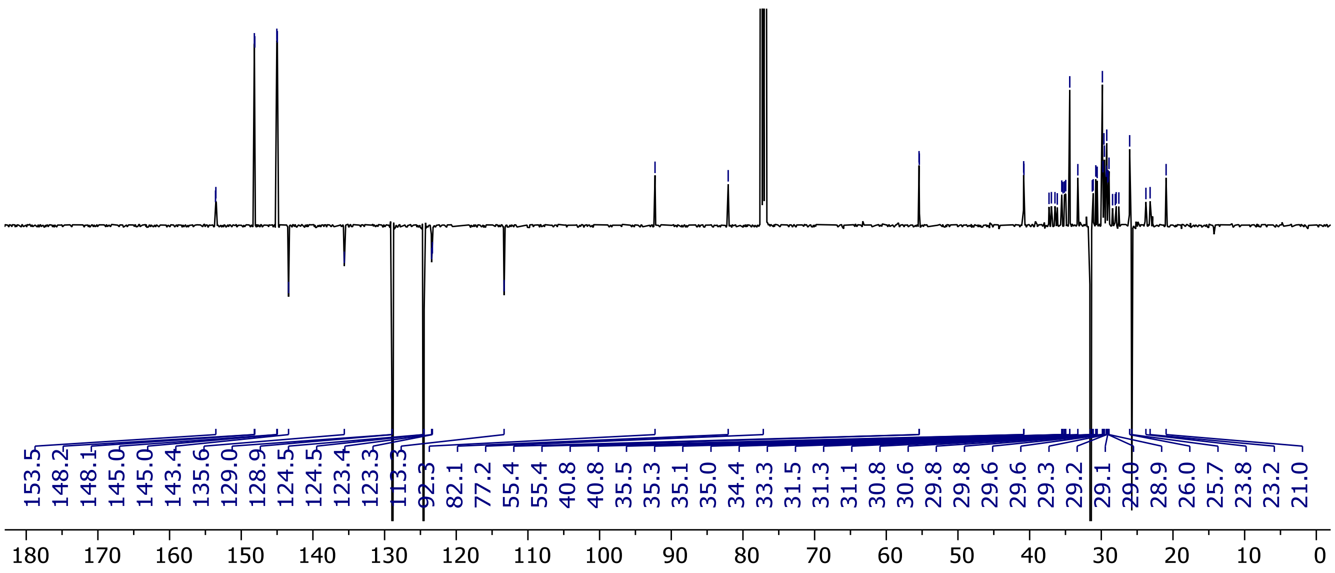

Figure S10. $^{13}C\{^{1}H\}$ APT NMR spectrum of **1** ($CDCl_3$, 126 MHz).

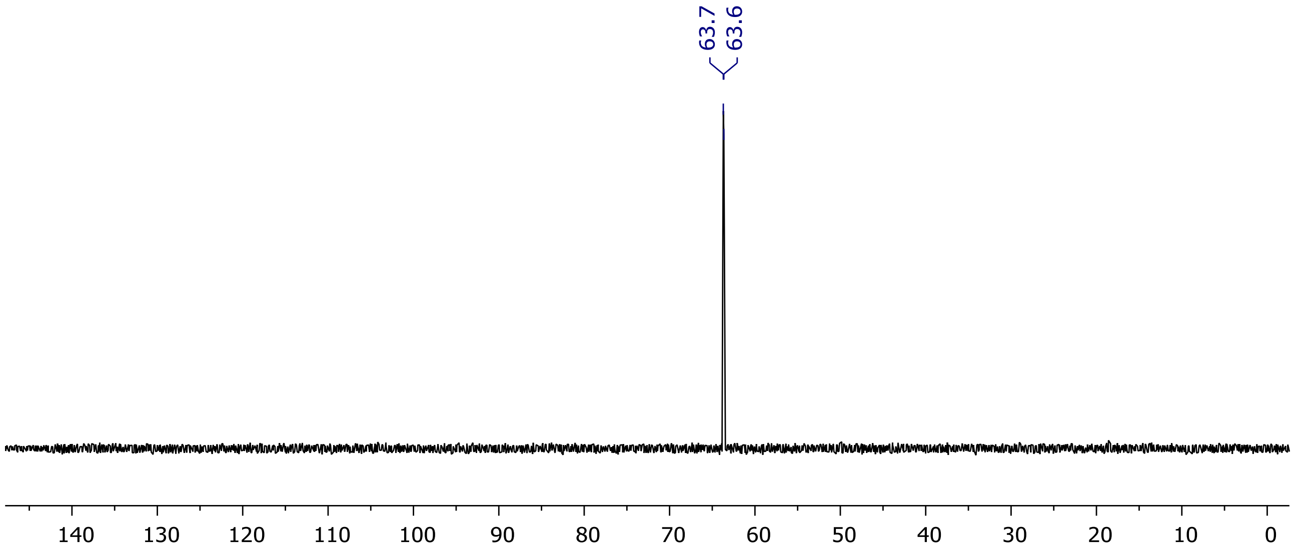

Figure S11. $^{31}P\{^{1}H\}$ NMR spectrum of **1** ($CDCl_3$, 162 MHz).

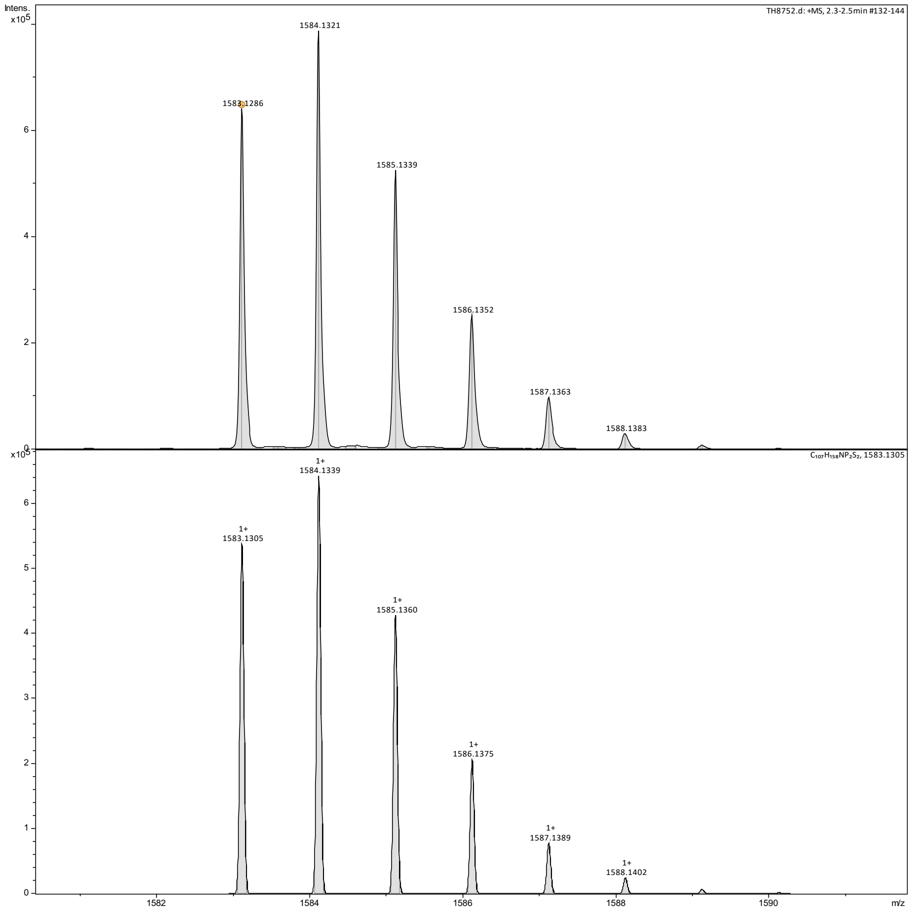

Figure S12. HR ESI-MS of **1** (top: observed, bottom: calcd).

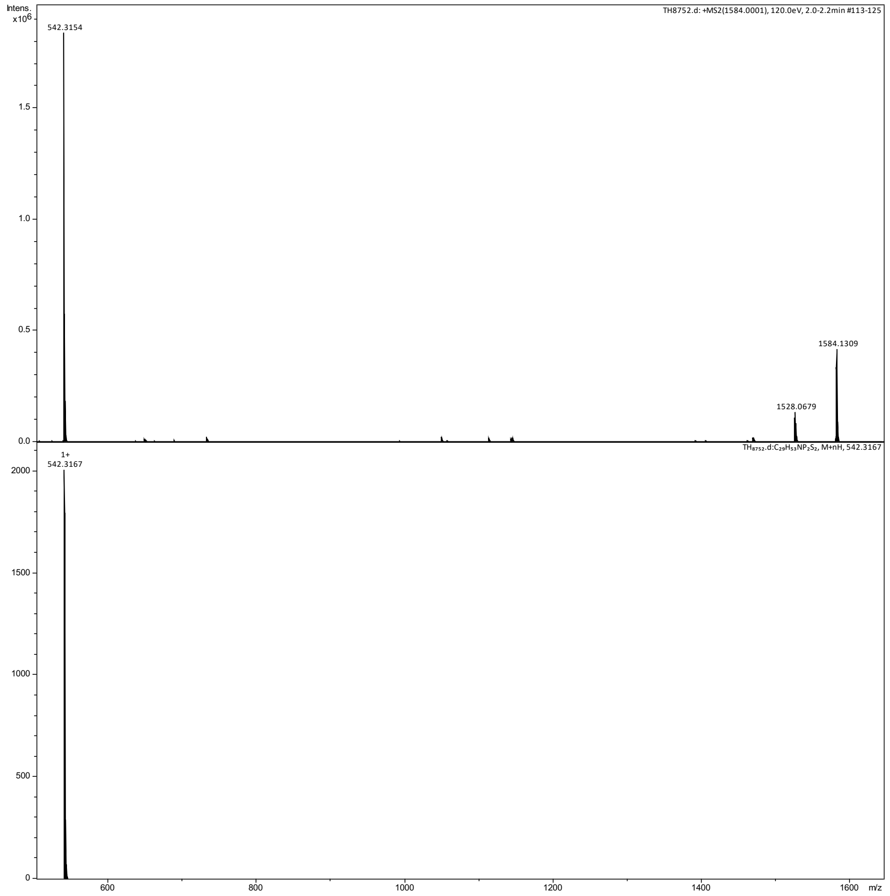

Figure S13. HR ESI-MS/MS (@ +1584) of **1** (top: observed, bottom: calcd).

4. Preparation of catenane 2

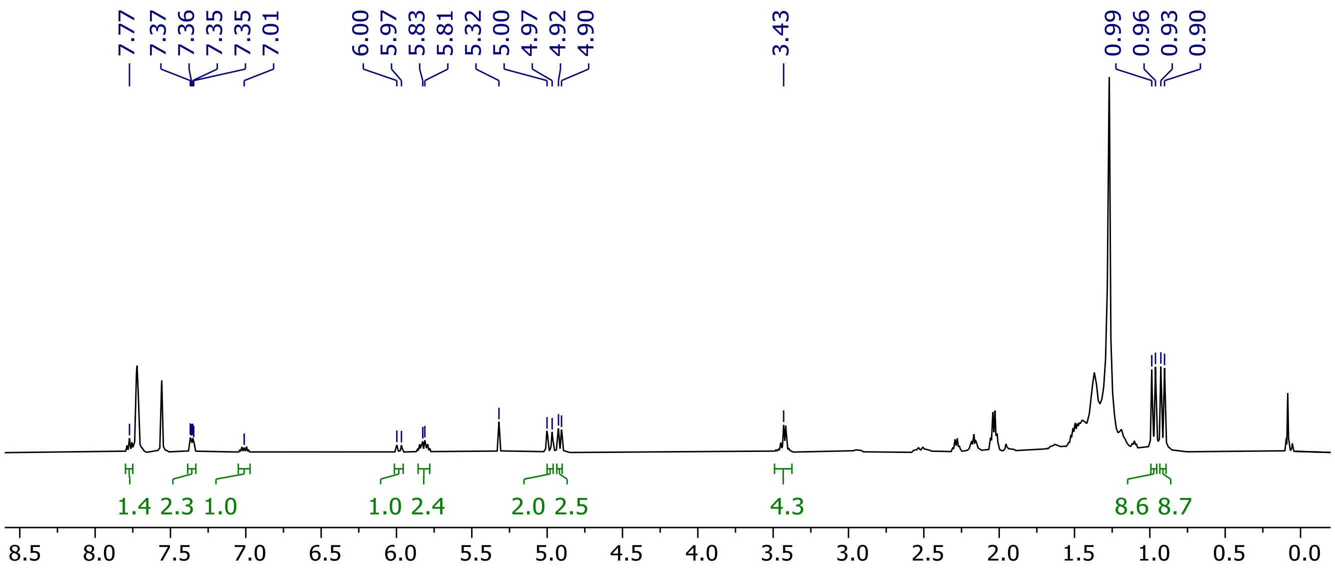

Figure S14. ^{1}H NMR spectrum of **4** (CD_2Cl_2, 500 MHz).

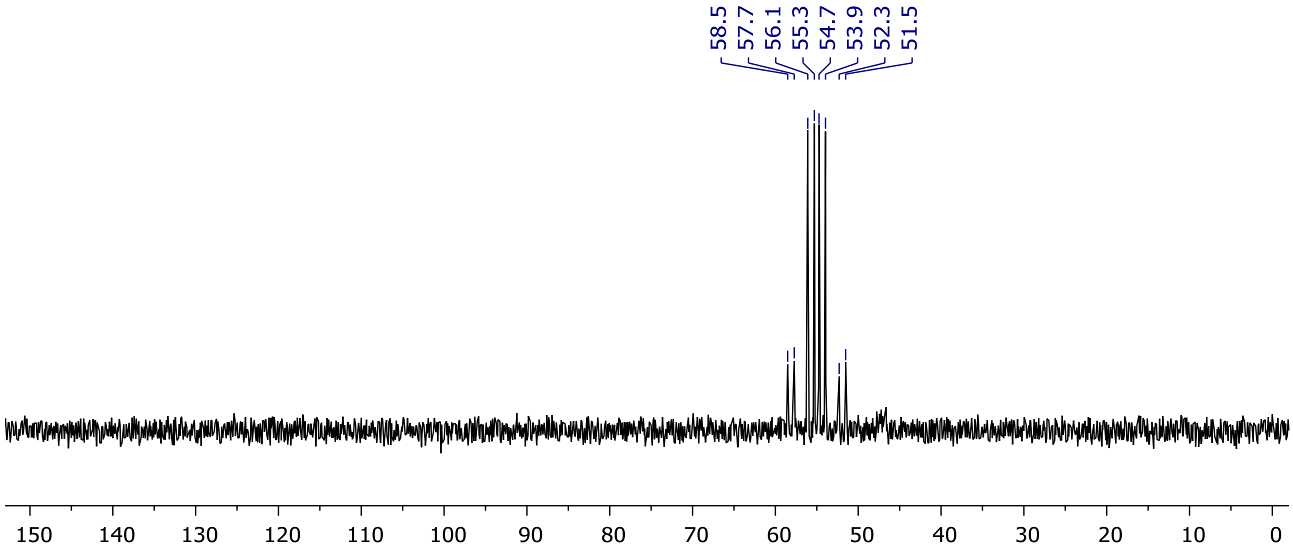

Figure S15. $^{31}P\{^{1}H\}$ NMR spectrum of **4** (CD_2Cl_2, 162 MHz).

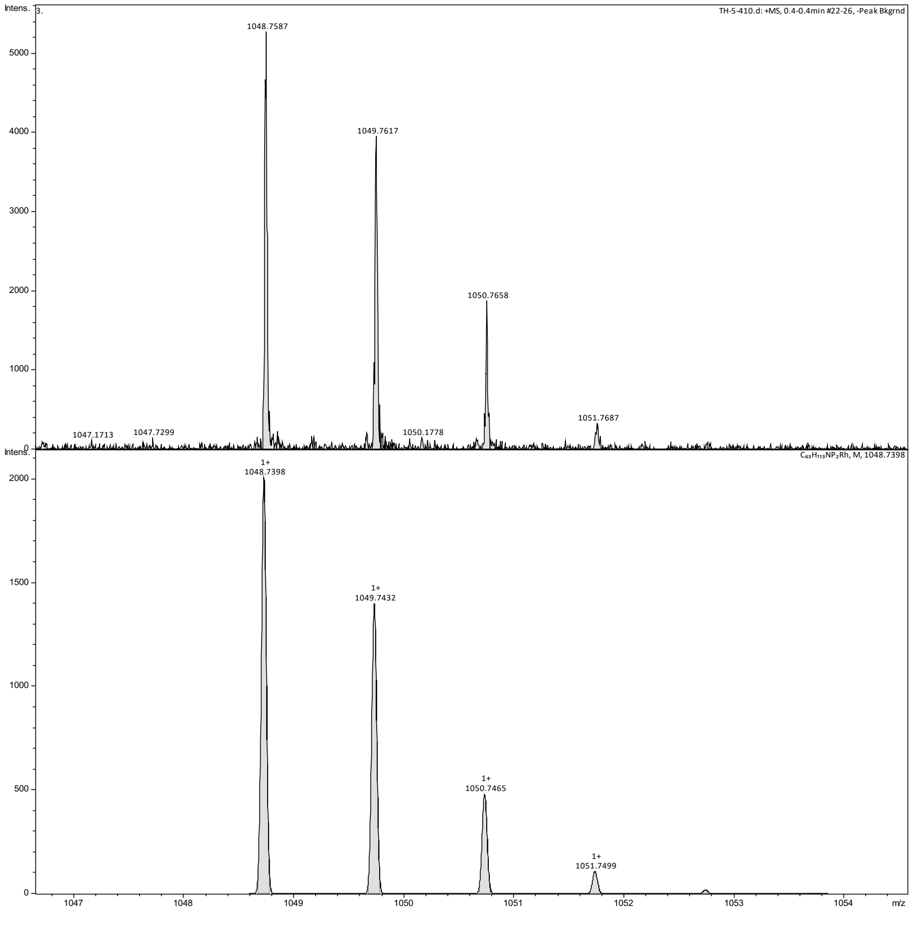

Figure S16. HR ESI-MS of **4** (top: observed, bottom: calcd).

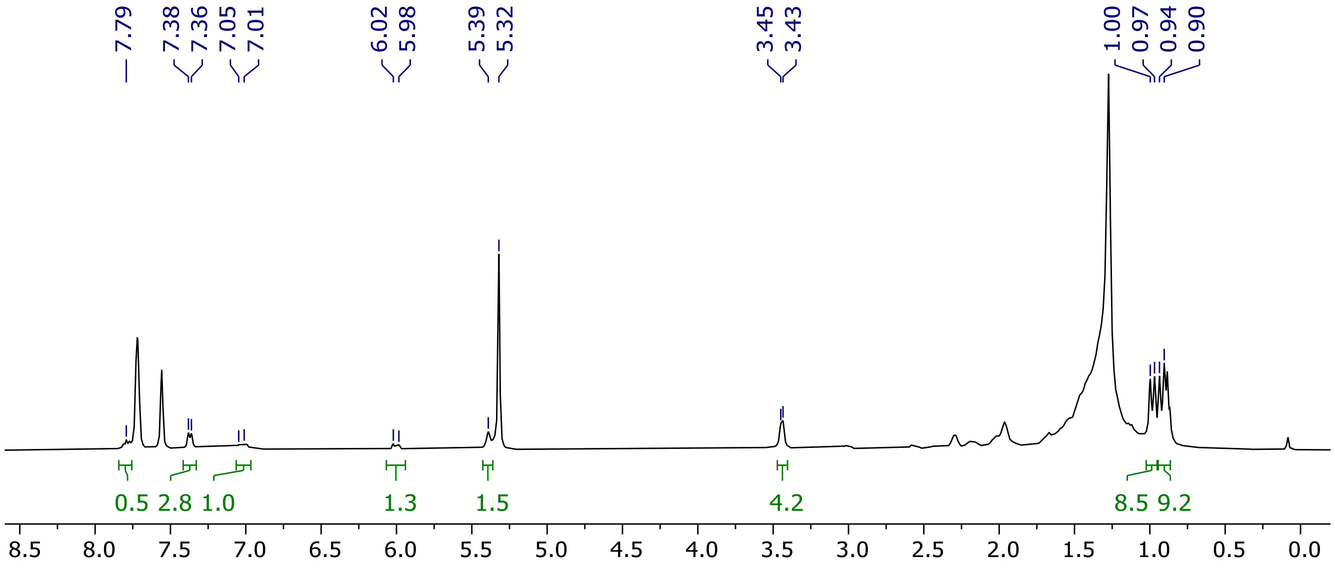

Figure S17. ^{1}H NMR spectrum of **5** (CD_2Cl_2, 400 MHz).

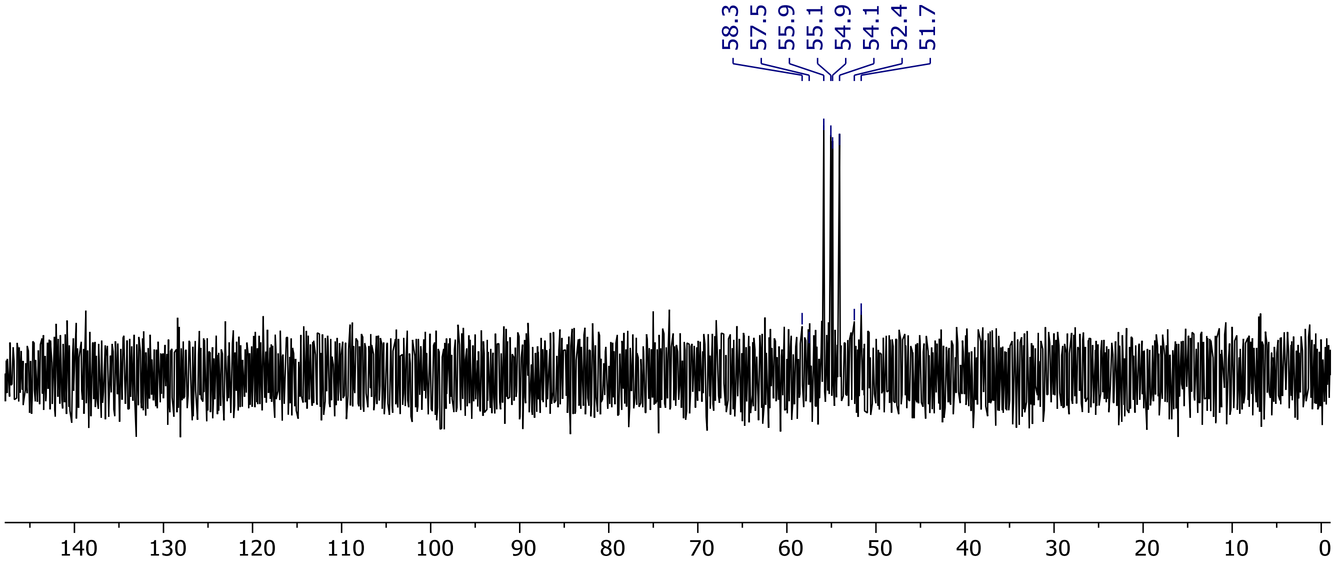

Figure S18. $^{31}P\{^{1}H\}$ NMR spectrum of **5** (CD_2Cl_2, 162 MHz).

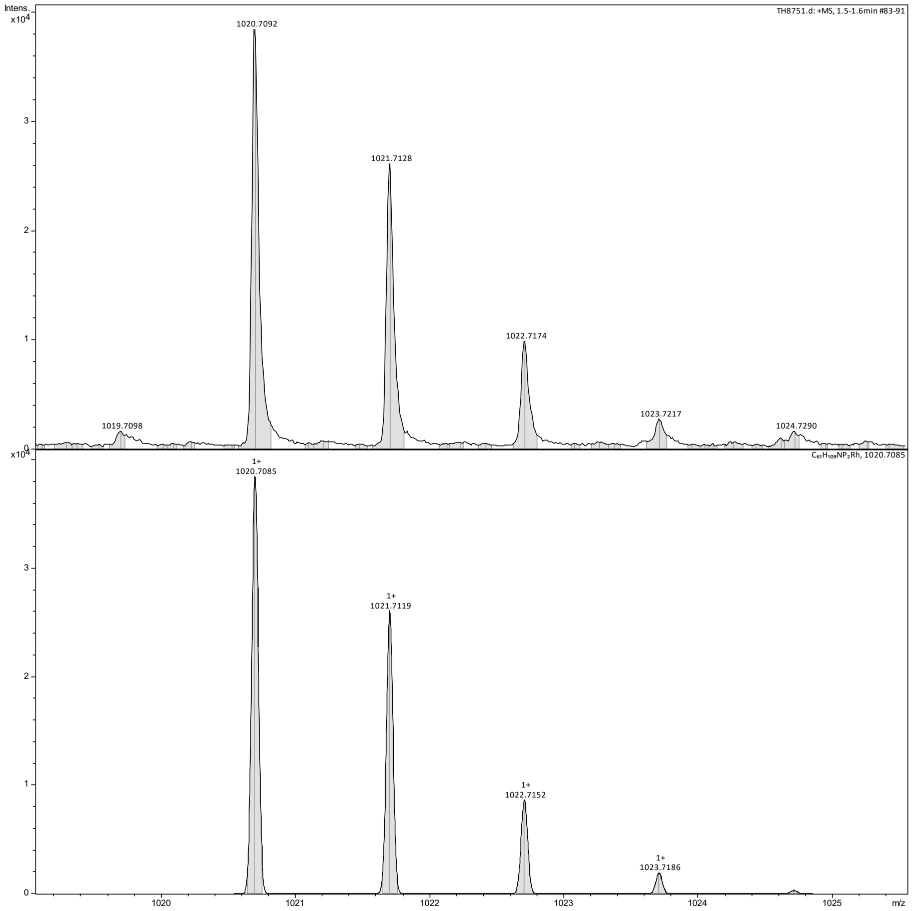

Figure S19. HR ESI-MS of **5** (top: observed, bottom: calcd).

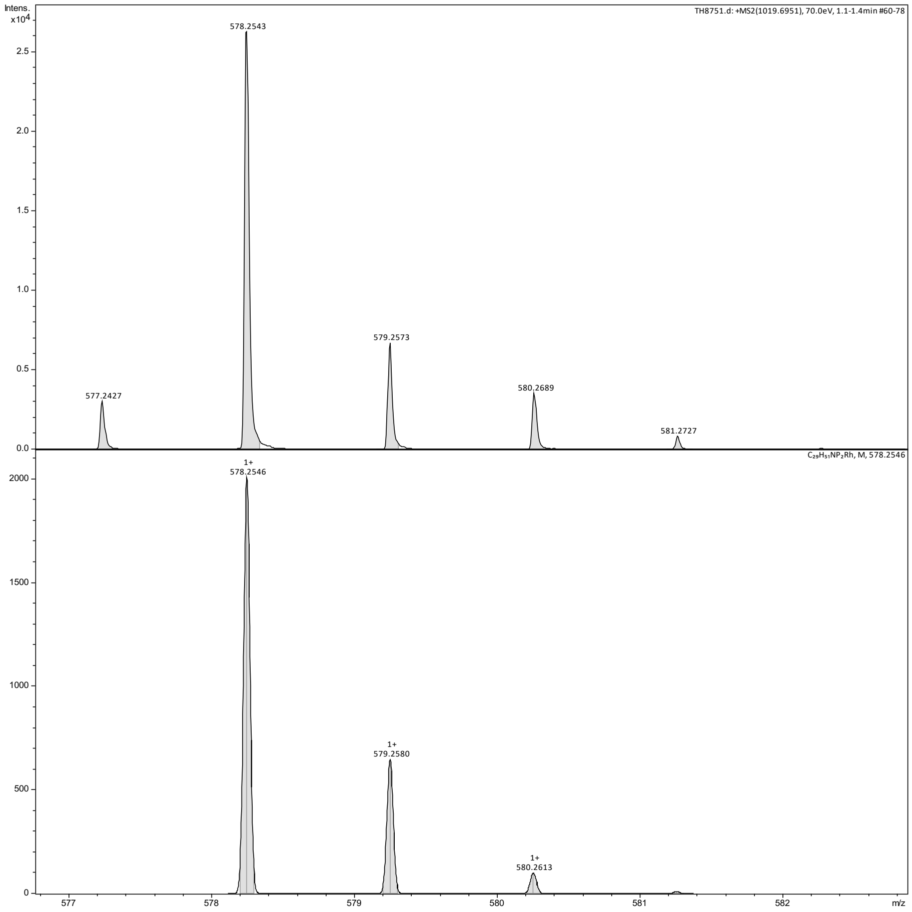

Figure S20. HR ESI-MS/MS (@ +1020) of **5** (top: observed, bottom: calcd).

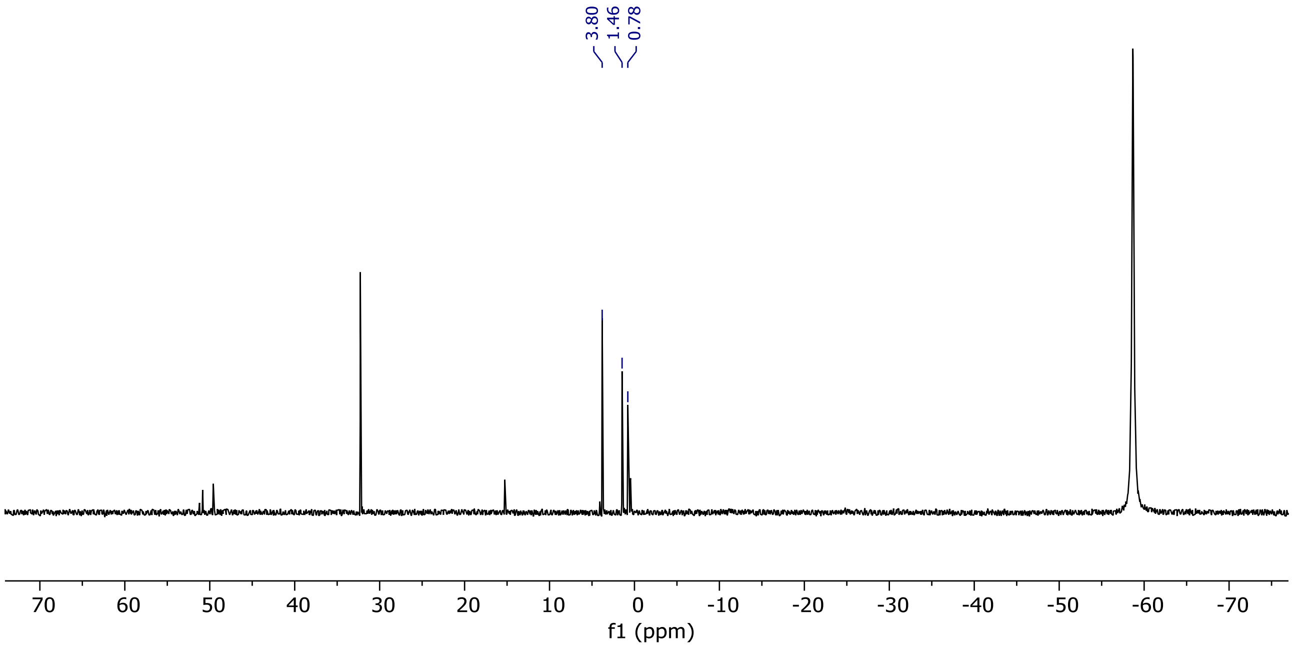

Figure S21. $^{31}P\{^1H\}$ NMR spectrum of **2′** (PhF, 162 MHz).

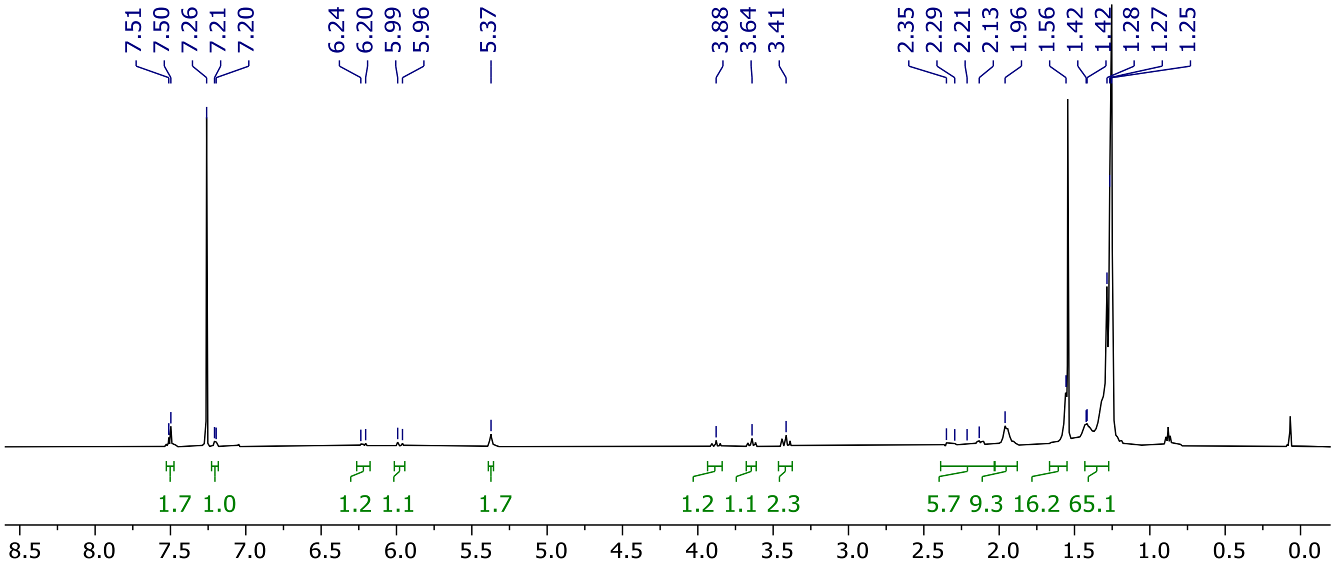

Figure S22. ^{1}H NMR spectrum of **2** ($CDCl_3$, 500 MHz).

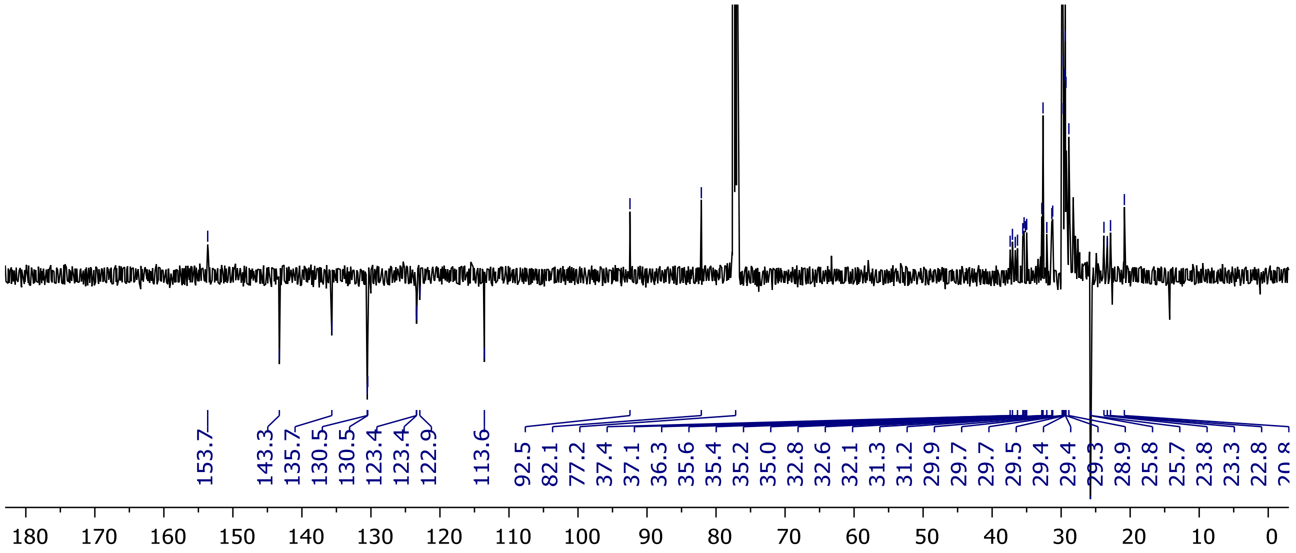

Figure S23. $^{13}C\{^{1}H\}$ APT NMR spectrum of **2** ($CDCl_3$, 126 MHz).

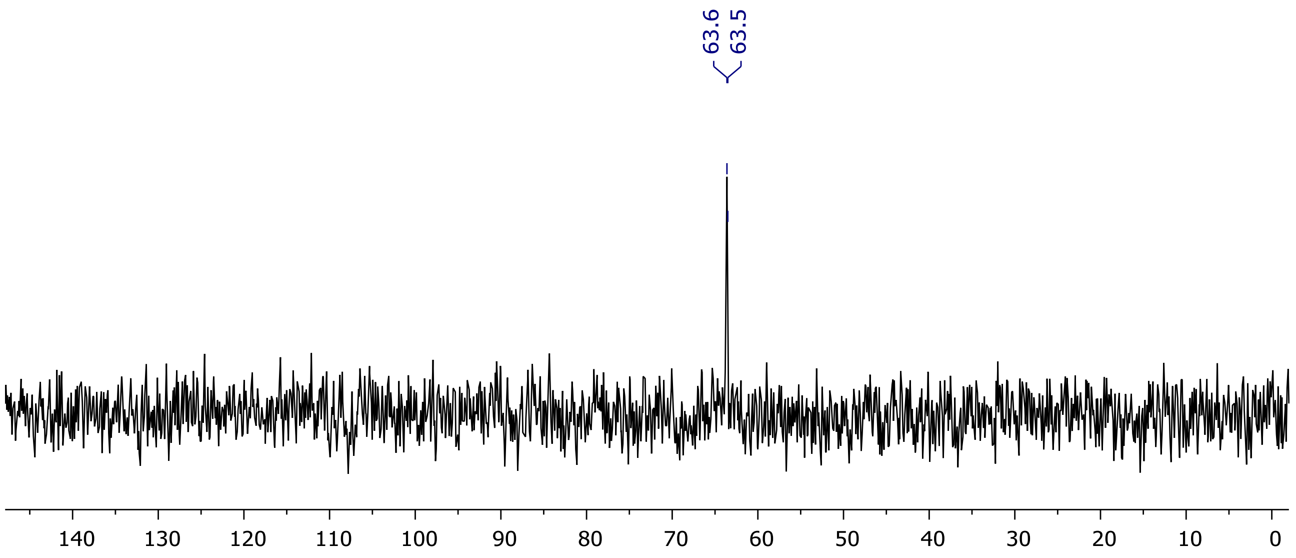

Figure S24. $^{31}P\{^{1}H\}$ NMR spectrum of **2** ($CDCl_3$, 162 MHz).

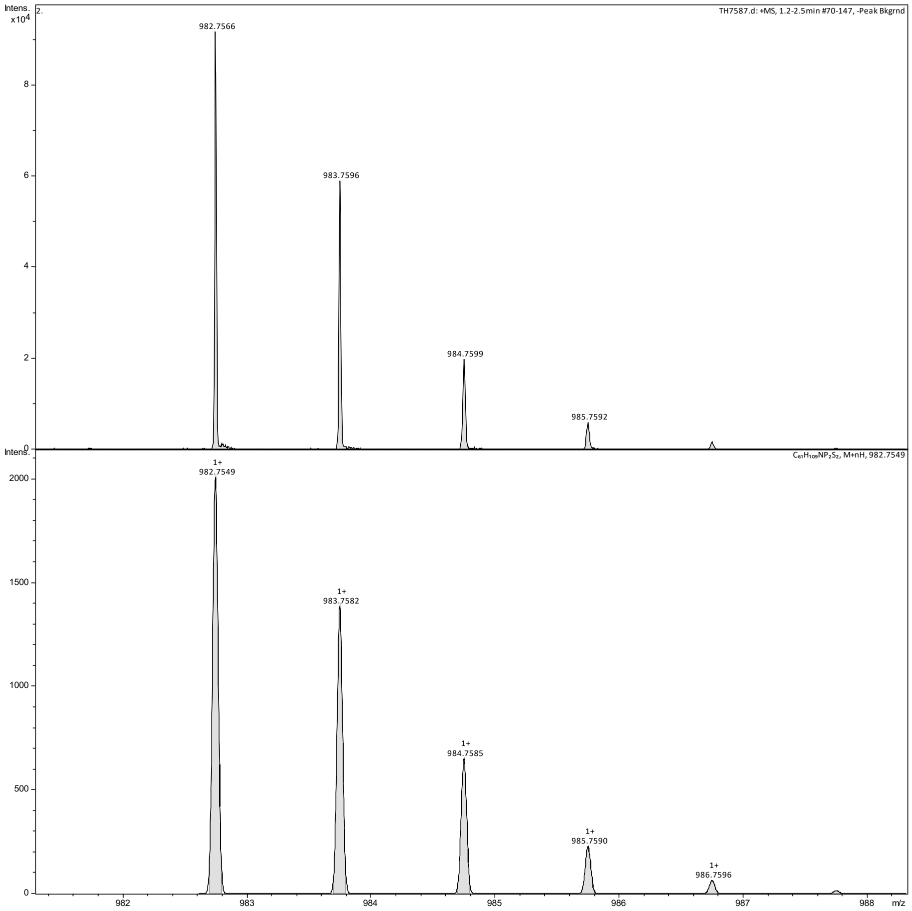

Figure S25. HR ESI-MS of **2** (top: observed, bottom: calcd).

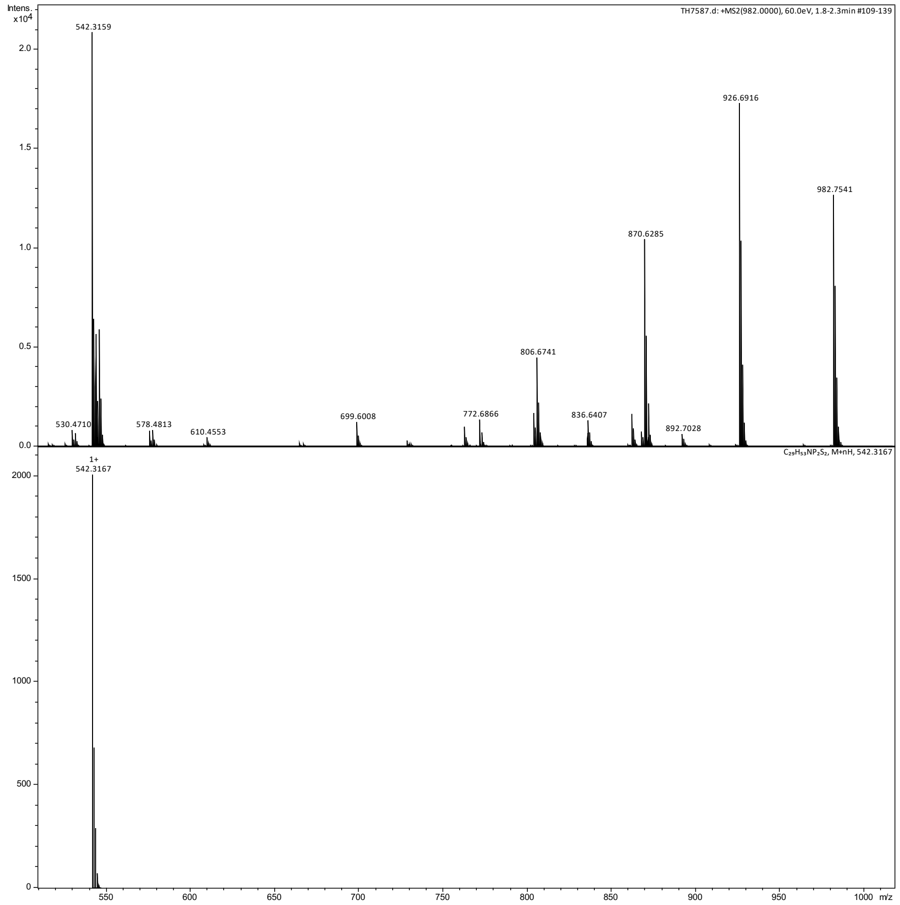

Figure S26. HR ESI-MS/MS (@ +982) of **2** (top: observed, bottom: calcd).

5. Preparation of PNP-14·2S

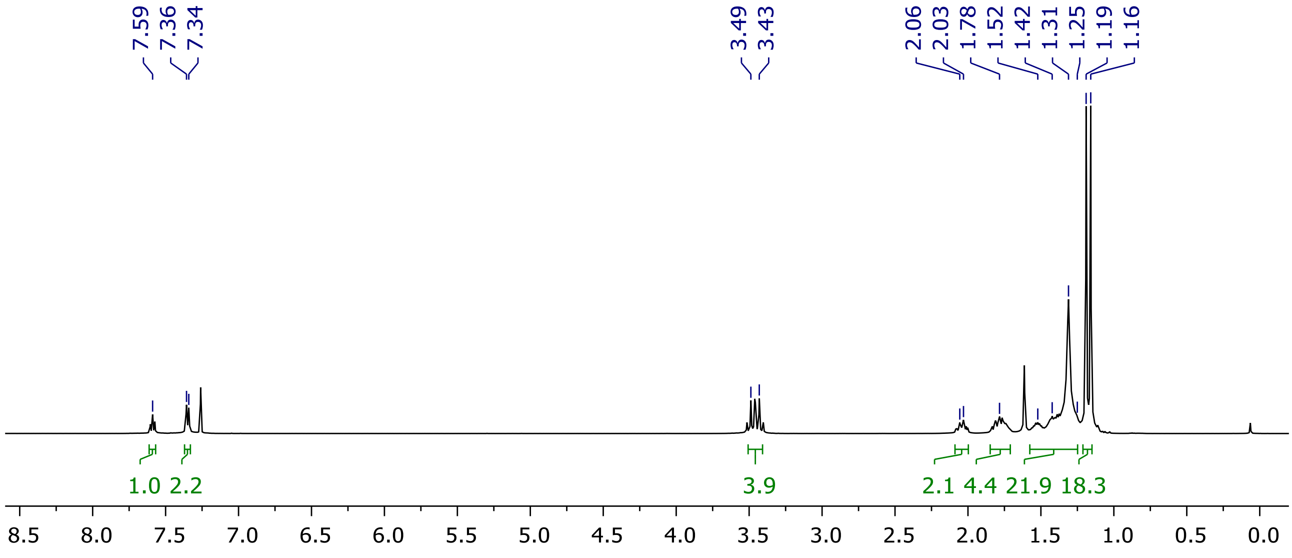

Figure S27. ^{1}H NMR spectrum of PNP-14·2S ($CDCl_3$, 500 MHz).

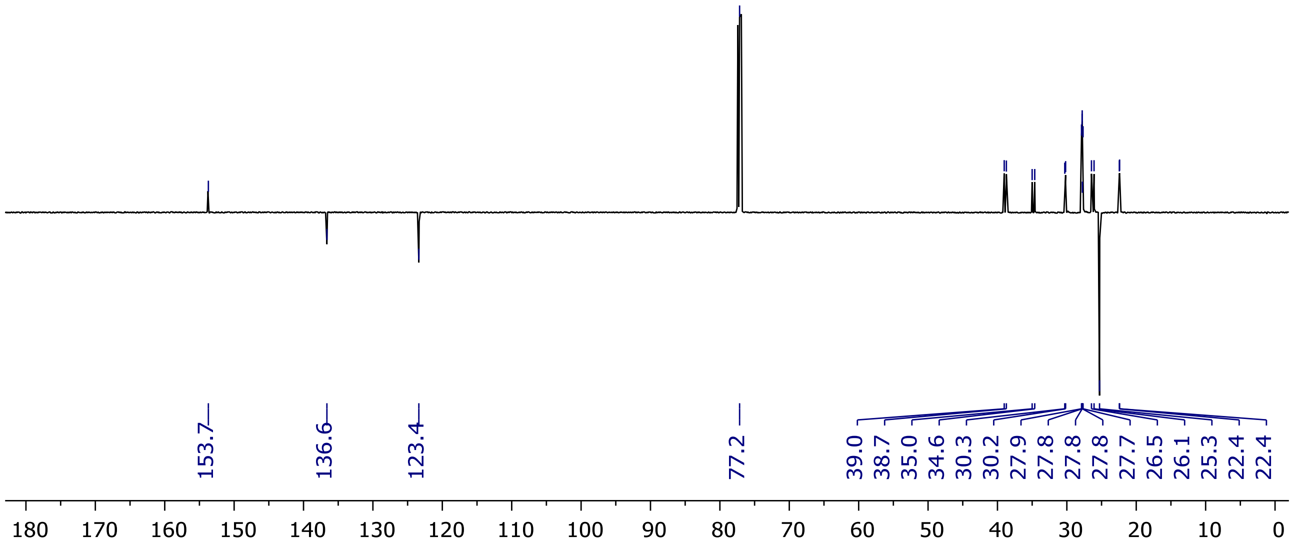

Figure S28. $^{13}C\{^{1}H\}$ APT NMR spectrum of PNP-14·2S ($CDCl_3$, 126 MHz).

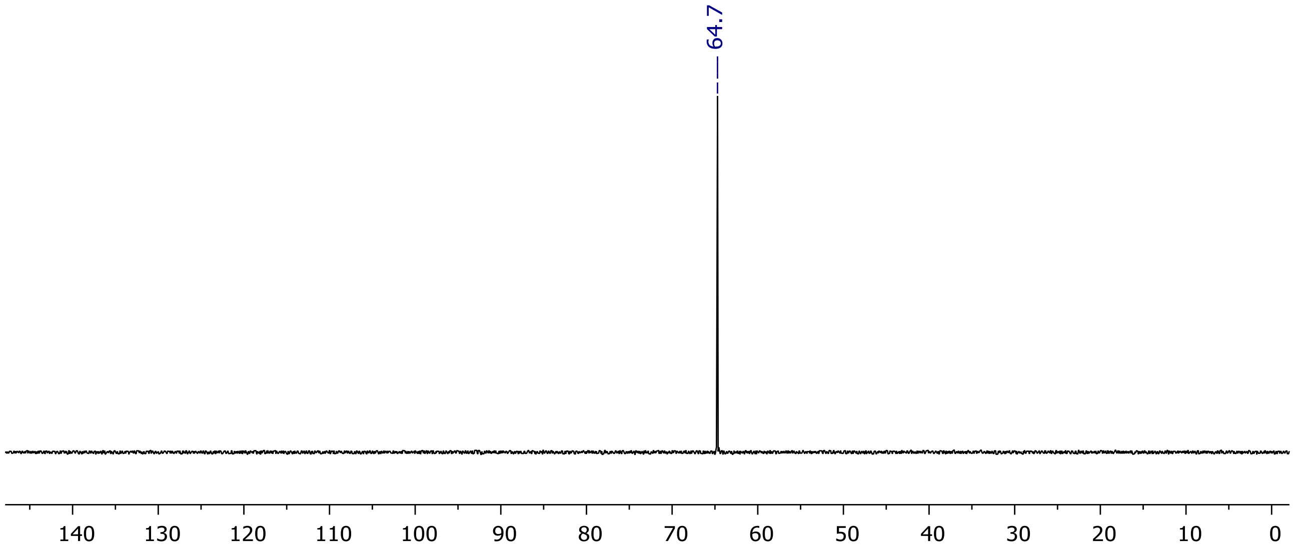

Figure S29. $^{31}P\{^{1}H\}$ NMR spectrum of PNP-14·2S ($CDCl_3$, 162 MHz).

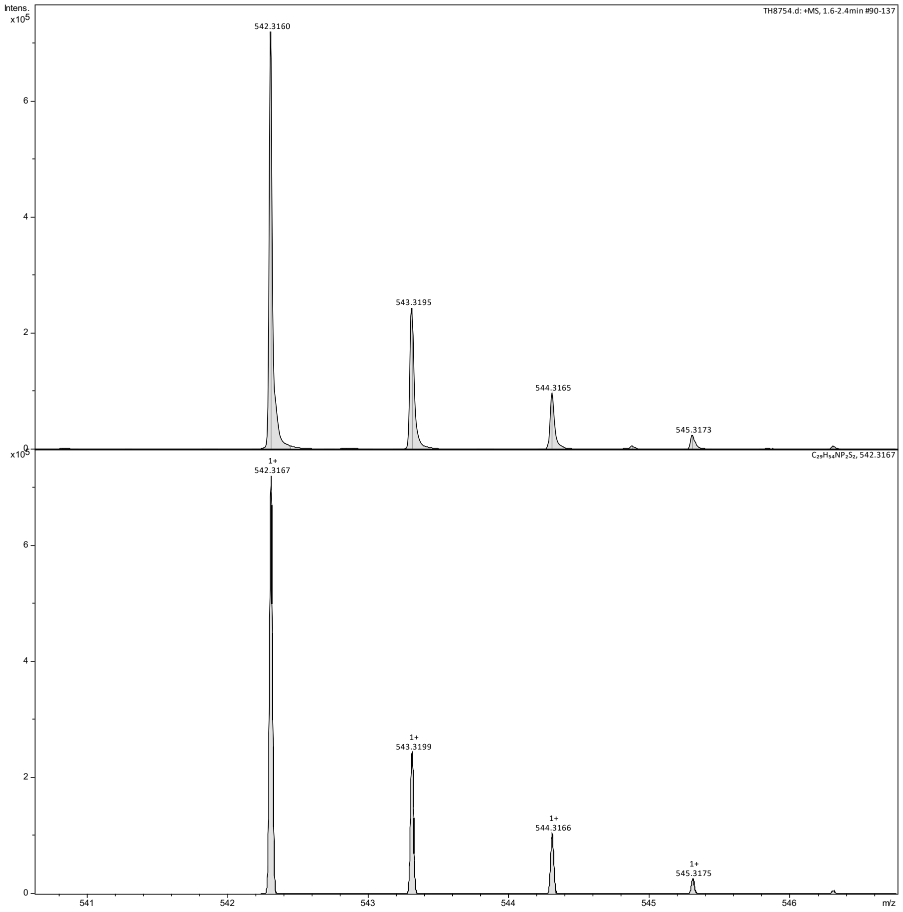

Figure S30. HR ESI-MS of PNP-14·2S (top: observed, bottom: calcd).

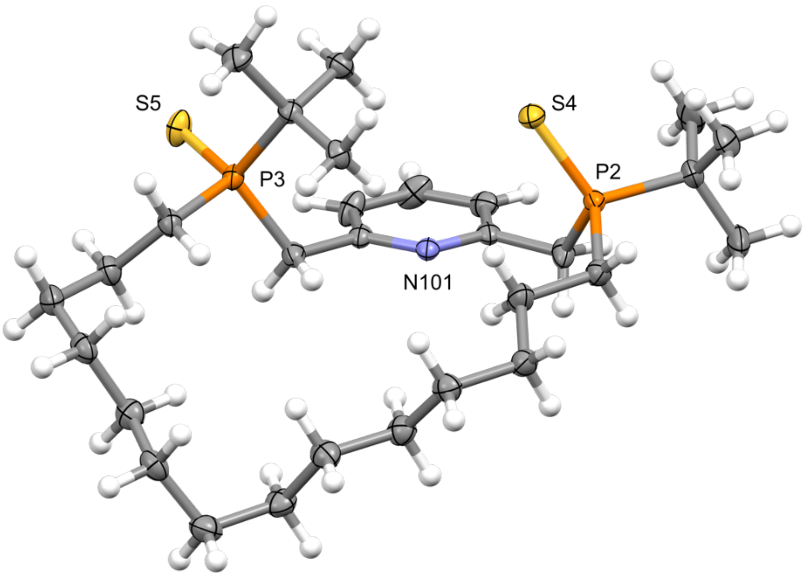

Figure S31. Solid-state structure of PNP-14·2S; thermal ellipsoids at 50% probability. Selected bond lengths (Å): P2–S4, 1.9564(4); P3–S5, 1.9629(4). CSD 2063081.

6. Comparison of NMR spectra

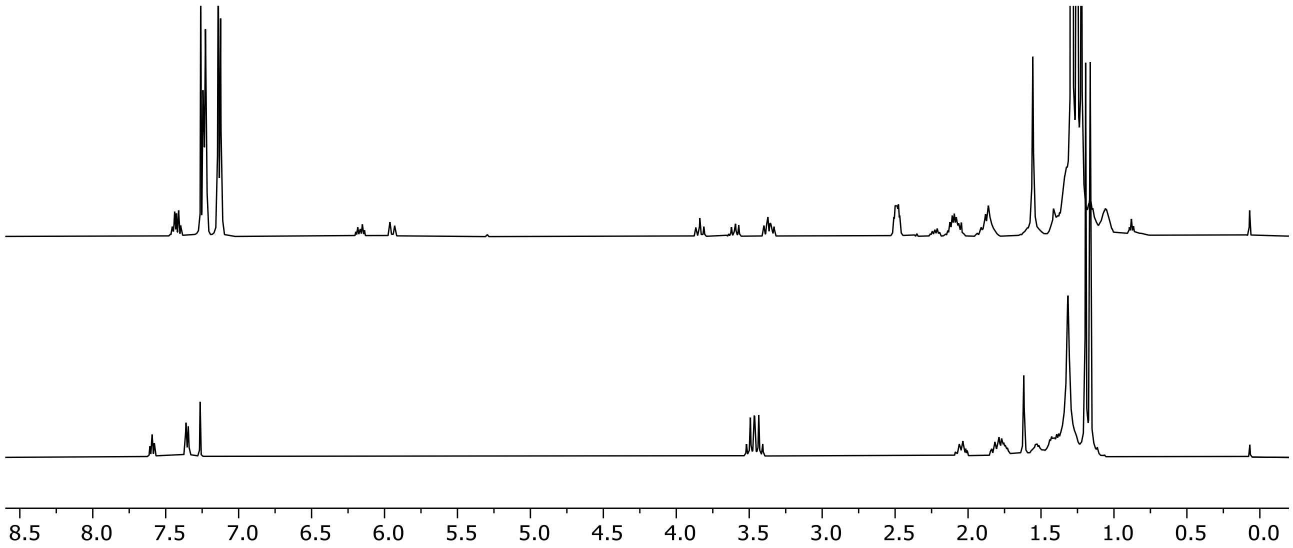

Figure S32. ^{1}H NMR spectra of **1** (top) and PNP-14·2S (bottom) ($CDCl_3$, 500 MHz).

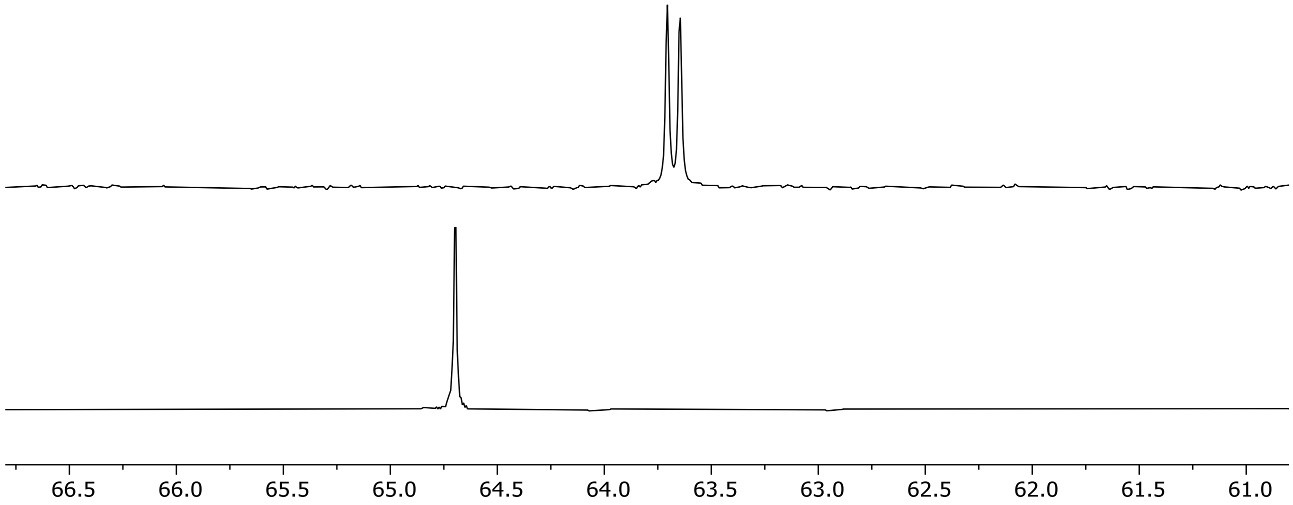

Figure S33. $^{31}P\{^{1}H\}$ NMR spectra of **1** (top) and PNP-14·2S (bottom) ($CDCl_3$, 162 MHz).

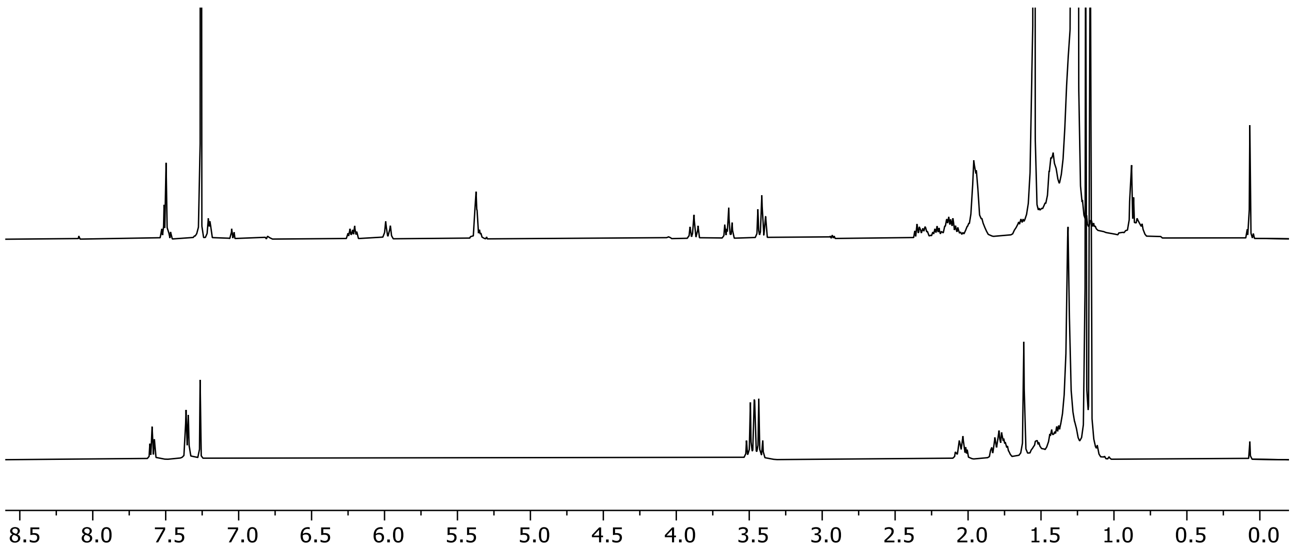

Figure S34. ^{1}H NMR spectra of **2** (top) and PNP-14·2S (bottom) ($CDCl_3$, 500 MHz).

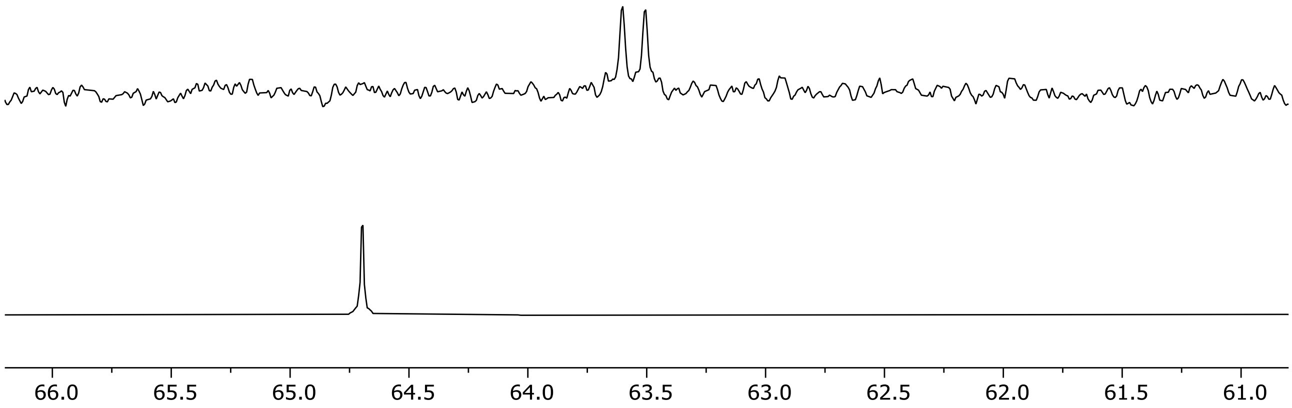

Figure S35. $^{31}P\{^{1}H\}$ NMR spectra of **2** (top) and PNP-14·2S (bottom) ($CDCl_3$, 162 MHz).
